# Genetic interactions among flowering loci influence phenology, vigour, and yield in chickpea

**DOI:** 10.1007/s11032-026-01701-5

**Published:** 2026-08-05

**Authors:** Raul Ortega Martinez, L. Lake, J. E. Hayes, J. L. Weller, R. French, V. O. Sadras

**Affiliations:** 1 School of Natural Sciences, University of Tasmania, Sandy Bay Campus, Hobart, Tasmania Australia; 2 ARC Centre of Excellence for Plant Success in Nature and Agriculture, University of Tasmania, Hobart, Tasmania Australia; 3 South Australian Research and Development Institute, Adelaide, South Australia Australia; 4 Waite Research Institute, School of Agriculture, Food and Wine, The University of Adelaide, Adelaide, South Australia Australia; 5 College of Science and Engineering, Flinders University, Adelaide, South Australia Australia; 6 Department of Primary Industries and Regional Development, Merredin, Western Australia Australia

**Keywords:** Breeding, Canopy, Genotype, Environment, Phenotype, Phenology

## Abstract

Flowering time and vegetative vigour are central adaptive traits in chickpea, shaping the balance between stress escape, biomass accumulation, and yield. Major phenology loci have been identified in this species, including *ELF3a*, *CaLG3b* and a cluster of *FT* homologues on chromosome 3 (*CaLG3a*), but their individual and interactive contributions to field adaptation remain unclear. Here, we developed near-isogenic lines (NILs) to dissect these loci under controlled conditions and across eight field environments in Southern and Western Australia. In a phytotron, NILs revealed strong photoperiod dependence: *ELF3a* suppressed flowering under short days, while early alleles at *CaLG3b* and the *FT* cluster promoted flowering under long days, with additive effects across loci. In the field, allelic substitutions consistently shifted phenology by a few days or nodes but rarely conferred direct yield benefits. Yield responses were variable and environment-dependent, with one NIL pair exhibiting a consistent penalty in environments with yields above 255 g m^− 2^. In contrast, canopy traits showed clearer divergence between NIL pairs, aligning with pleiotropic effects of the *FT* cluster. These results highlight the utility of NILs for attributing modest but adaptive phenological shifts to defined genomic regions, and suggest that the primary breeding value of these loci lies in fine-tuning crop development to local stress patterns rather than delivering robust yield gains. Stacking early and late alleles may offer breeders a genetic tool to broaden adaptive flexibility, while yield improvement will likely require integration with physiological traits related to resource capture, efficiency and higher reproductive allocation.

**Supplementary Information:**

The online version contains supplementary material available at 10.1007/s11032-026-01701-5.

## Introduction

Phenological development is central to both plant fitness in nature and seed production of annual crops (Burton et al. 2025; Darwin 1859; Flohr et al. 2018). Knowledge of the genetic basis of flowering time and related phenology traits in legumes has grown in recent decades, aided by a combination of comparative studies with model species, classical genetic analyses, and broader association studies conducted mostly in pea (*Pisum sativum*), barrel medic (*Medicago truncatula*), and soybean (*Glycine max*), with minor contributions from other species like chickpea (*Cicer arietinum*), lentil (*Lens culinaris*) and common bean (*Phaseolus vulgaris*) (Lin et al. 2021; Luo et al. 2022; Weller and Macknight 2018, 2020; Weller and Ortega 2015).

In chickpea, the focus of this study, early flowering is often important to avoid drought in the critical period around podding, particularly in Mediterranean and South Asian cropping systems, and has therefore been a consistent target of selection during domestication and breeding (Berger et al. 2006; Kumar and Abbo 2001). This agronomic relevance has driven the identification of genetic regions recurrently associated with phenological traits (Mallikarjuna et al. 2017; Weller and Ortega 2015; Perez-Rial et al. 2024). Among these, only the classical *Early flowering 1* (*Efl1*) locus has been molecularly characterized. *Efl1* encodes for *CaELF3a*, an ortholog of *Arabidopsis EARLY FLOWERING 3 (ELF3)*, a circadian-clock component that represses flowering when photoperiodic requirements are not met (Kumar et al. 1999; Liu et al. 2001; Ridge et al. 2017). The photoperiod-insensitive early flowering phenotype of chickpea *efl1* mutants suggest a conserved repressive function for *CaELF3a* in the chickpea flowering pathway (Ridge et al. 2017).

Beyond *Efl1*, a central region on chromosome 3 has emerged as a recurrent hotspot for flowering time variation across diverse genetic backgrounds (Cobos et al. 2009; Daba et al. 2016; Hamwieh et al. 2013; Hossain et al. 2010; Mallikarjuna et al. 2017; Rehman et al. 2011; Samineni et al. 2015; Carmona et al. 2026), suggesting that this might be a widespread source of flowering time variation within chickpea germplasm. Subsequent studies resolved this region into two distinct loci (Atieno et al. 2021; Ortega et al. 2019). The first (herein referred to as *CaLG3a*) co-locates with a cluster of three *FT* genes (namely *FTa1–FTa2* and *FTc*), which have been proposed as the most likely candidates based on QTL mapping, sequence variation and expression analysis (Ortega et al. 2019). *FT* genes encode mobile floral integrators that function downstream of photoperiod and vernalisation pathways, triggering floral initiation at the shoot apex (Wickland and Hanzawa 2015; Gao et al. 2026). Deregulation or gain-of-function alleles at this locus have been repeatedly associated with early flowering, particularly under short-days, as well as with other agronomically relevant architectural traits such as reduced branching and erect growth habit (Ortega et al. 2019). Moreover, the early flowering *CaLG3a* allele also associates with high vigour in cultivated germplasm (Nguyen et al. 2022). By contrast, the genetic factors that underlie the second locus (*CaLG3b*) are currently unknown. The genomic interval within identified flanking markers for *CaLG3b* remains large and poorly defined, hindering progress towards gene elucidation.

Although *CaELF3a*, *CaLG3a*, and *CaLG3b* have been individually characterized, their potential interactions and contributions to crop performance have not been investigated. Understanding how these loci interact could provide new insights into the genetic architecture of flowering time and its associated traits in chickpea. Near-isogenic lines (NILs) are ideal for this purpose, as they share a common genetic background, differing in only a few small parts of the genome that include the region associated with a trait. They can be produced using several methods. Two contrasting genotypes may be crossed, followed by recurrent back-crossing and selection (genetically or phenotypically) through a number of generations, to develop related backcross-derived lines with contrasting alleles for a target phenotype. An example of this is Tamaroi/Tamaroi *Nax2* high- and low-sodium accumulating durum wheat near-isogenic pairs, used to explore aspects of salinity tolerance in cereals (Munns et al. 2012). Alternatively, the residual heterozygosity in Recombinant Inbred Lines (RILs) derived from bi-parental populations used for genetic mapping and trait dissection can be exploited, by selecting and progressing RILs still displaying heterozygosity across a region of interest at an advanced generation. Progeny can be selected, genetically or phenotypically, to carry contrasting alleles. The method has previously been described as heterogeneous inbred family (HIF) analysis (Tuinstra et al. 1997).

For this study, we developed sets of NILs from a chickpea RIL population *Rupali* × *Genesis836* (Atieno et al. 2021), with varying allelic combinations for *CaLG3a*, *CaLG3b* and *CaELF3a*. By combining experiments under controlled conditions and field environments, we aimed to (i) quantify the individual and interactive effects of these loci on phenological development, (ii) evaluate the extent to which allelic effects observed under controlled conditions translate to agronomically relevant environments, and (iii) assess their contribution to variation in vigour and grain yield, examining potential trade-offs between these traits. Through this approach, we provide new insights into the genetic architecture of phenology in chickpea and its implications for breeding, and propose a conceptual model of these interactions.

## Materials and methods

### NIL development

Material from a *Rupali* × *Genesis836* population described in Atieno et al. (2021) was used for the development of the near-isogenic lines used in this study. *Genesis836* carries the early allele at the *CaLG3b* locus and bears a deletion in the *CaLG3a*/*FT* cluster that was previously associated with flowering time and plant vigour (Nguyen et al. 2022; Ortega et al. 2019), while cv. *Rupali* carries the early *elf3a* mutant allele (Ridge et al. 2017). For clarity, *Genesis836* alleles for these loci will be referred to as *LG3b*^*Early*^, *LG3a*^*del*^ and *ELF3a*^*WT*^, while alleles from *Rupali* will be referred as *LG3b*^*Late*^, *LG3a*^*WT*^ and *elf3a* (Table 1).

With emphasis on the *CaLG3b* and *CaLG3a* regions, potentially useful recombinant inbred lines (RILs) that still retained heterozygosity across these regions were first identified by inspection of the *Rupali* × *Genesis836* genetic map (Atieno et al. 2021). Advanced generations (F_5_-F_6_, depending on seed availability) were grown for each selected RIL. DNA was extracted from leaf tissue, and genotypes at each region were determined with Kompetitive Allele Specific Polymorphism (KASP™) markers (Table S1). Identification of homozygous alleles within a RIL provided immediate NIL pairs, while in some cases heterozygotes individuals were progressed further for additional screening. Markers flanking a broad interval were chosen for genotyping at *CaLG3b*, alongside markers for *CaLG3a* and *CaELF3a*.

Where possible, NIL pairs with common phenology allele combinations were generated in different RIL backgrounds to minimise association of phenotype with other segregating genomic regions. Genome-wide marker information of RIL backgrounds for each NIL pair is provided in Table S2.

**Table 1:** Genotype of NIL pairs derived from a *Rupali* x *Genesis836* chickpea RIL population phenotyped in controlled environment (CEF), field or both. In the field, pairs segregating for *CaLG3a* were named as Vigour (Vig) NILs while those segregating for *CaLG3b* were named as Flowering time (FT) NILs

NIL name	Identifier	Environment	CaELF3a	CaLG3b	CaLG3a
**Ca3a NILs / Vigour NILsLG**					
CaLG3a1 (Vig NIL 1)	CaLG3a1_G1 (Vig NIL1G)	CEF + Field	*ELF3a* ^*WT*^	*LG3b* ^*Early*^	*LG3a* ^*Del*^
	CaLG3a1_R1 (Vig NIL1R)				*LG3a* ^*WT*^
	CaLG3a1_G2	CEF			*LG3a* ^*Del*^
	CaLG3a1_R2				*LG3a* ^*WT*^
CaLG3a2 (Vig NIL 2)	CaLG3a2_G	CEF	*elf3a*	*LG3b* ^*Late*^	*LG3a* ^*Del*^
	CaLG3a2_R				*LG3a* ^*WT*^
	Vig NIL2G	Field			*LG3a* ^*Del*^
	Vig NIL2R				*LG3a* ^*WT*^
CaLG3a3 (Vig NIL 3)	CaLG3a3_G1	CEF	*ELF3a* ^*WT*^	*LG3b* ^*Late*^	*LG3a* ^*Del*^
	CaLG3a3_R1				*LG3a* ^*WT*^
	CaLG3a3_G2 (Vig NIL3G)	CEF + Field			*LG3a* ^*Del*^
	CaLG3a3_R2 (Vig NIL3R)				*LG3a* ^*WT*^
CaLG3a4 (Vig NIL 4)	CaLG3a4_G1	CEF	*elf3a*	*LG3b* ^*Early*^	*LG3a* ^*Del*^
	CaLG3a4_R1				*LG3a* ^*WT*^
	CaLG3a4_G2 (Vig NIL4G)	CEF + Field			*LG3a* ^*Del*^
	CaLG3a4_R2 (Vig NIL4R)				*LG3a* ^*WT*^
**CaLG3b** ** NLs / FloIwering time NILs**					
CaLG3b1 (FT NIL 1)	CaLG3b1_G	CEF	*elf3a*	*LG3b* ^*Early*^	*LG3a* ^*WT*^
	CaLG3b1_R			*LG3b* ^*Late*^	
	FT NIL1G	Field		*LG3b* ^*Early*^	
	FT NIL1R			*LG3b* ^*Late*^	
CaLG3b2 (FT NIL 2)	CaLG3b2_G (FT NIL2G)	CEF + Field	*ELF3a* ^*WT*^	*LG3b* ^*Early*^	*LG3a* ^*Del*^
	CaLG3b2_R (FT NIL2R)			*LG3b* ^*Late*^	
CaLG3b3 (FT NIL 3)	FT NIL3G	Field	*ELF3a* ^*WT*^	*LG3b* ^*Early*^	*LG3a* ^*Del*^
	FT NIL3R			*LG3b* ^*Late*^	
CaLG3b4 (FT NIL 4)	FT NIL4G	Field	*ELF3a* ^*WT*^	*LG3b* ^*Early*^	*LG3a* ^*Del*^
	FT NIL4R			*LG3b* ^*Late*^	
CaLG3b5	CaLG3b5_G1	CEF	*elf3a*	*LG3b* ^*Early*^	*LG3a* ^*WT*^
	CaLG3b5_R1			*LG3b* ^*Late*^	
	CaLG3b5_G2			*LG3b* ^*Early*^	
	CaLG3b5_R2			*LG3b* ^*Late*^	
CaLG3b6	CaLG3b6_G1	CEF	*ELF3a* ^*WT*^	*LG3b* ^*Early*^	*LG3a* ^*WT*^
	CaLG3b6_R1			*LG3b* ^*Late*^	
	CaLG3b6_G2			*LG3b* ^*Early*^	
	CaLG3b6_R2			*LG3b* ^*Late*^	
CaLG3b7	CaLG3b7_G1	CEF	*elf3a*	*LG3b* ^*Early*^	*LG3a* ^*Del*^
	CaLG3b7_R1			*LG3b* ^*Late*^	
	CaLG3b7_G2			*LG3b* ^*Early*^	
	CaLG3b7_R2			*LG3b* ^*Late*^	

### Experiment 1: developmental responses of NILs to photoperiod in controlled environment

Fifteen NIL pairs segregating for either *CaLG3b* or *CaLG3a* were evaluated under contrasting photoperiod in controlled facilities (Table 1). A total of 480 plants (4–8 replicates per line and treatment), including parental lines *Rupali* and *Genesis836*, were grown in 140 mm pots filled with potting mix supplemented with controlled-release fertiliser. Plants were exposed to short-day (SD; 8 h light:16 h dark) or long-day (LD; 16 h light:8 h dark) as follows: SD plants received 8 h of natural daylight, and were then transferred to a dark phytotron for 16 h. LD plants received 8 h of natural daylight and were then transferred to a phytotron where the photoperiod was extended another 8 h using Heliospectra ELIXIA LED growth lights (https://www.heliospectra.com), featuring a red (R; 650–670 nm) to far-red (FR; 720–740 nm) ratio (Smith 1982) of 0.93 ± 0.003 representing a flowering-inductive long day, and a very low photosynthetically active radiation (PAR; 36 ± 0.03 µmol m^–2^ s^–1^) to avoid a confounding effect between daylength as a signal and resource availability. The spectral photon flux and PAR were measured using an STS-VIS microspectrometer (Ocean Insight, https://www.oceaninsight.com). Temperature was maintained at 15 °C in the phytotron, with external conditions monitored throughout (Fig. S1).

**Table 2:** Phenology traits measured in controlled environment

Trait		Description
DTE	Days to emergence	Number of days from sowing to seedling emergence
NFI	Node of flower initiation	Number of nodes to the initiation of the first floral structure, whether a fully developed flower or an aborted bud
NFD	Node of flower development	Number of nodes to the first developed flower
DFI	Days to flower initiation	Number of days from seedling emergence to the appearance of the first floral structure, whether a fully developed flower or an aborted bud
DFD	Days to flower development	Number of days from seedling emergence to the appearance of the first developed flower
NPD	Node of pod development	Number of nodes to the first fully viable pod
DPD	Days to pod development	Number of days from seedling emergence to the appearance of an early pod structure that will become a fully viable pod.
NFGAP	Gap in flowering node	Difference, in nodes, between flower initiation and flower development (NFD-NFI)
DFDELAY	Delay in flowering time	Number of days between flower initiation and flower development (DFD-DFI)
NPGAP	Gap in podding node	Difference, in nodes, between flower development and pod development (NPD-NFD)
DPDELAY	Delay in podding time	Number of days between flower development and pod development (DPD-DFD)

Eleven traits associated with phenology were recorded in each photoperiod (Table 2). Additionally, photoperiod sensitivity was calculated as the difference in time to flowering (DFD) between short and long days. For a more accurate determination of node number, plants were debranched weekly.

### Experiment 2: development, growth and yield of NILs in the field

#### Experiment

Crops were grown in eight rainfed environments resulting from the combination of locations, years and sowing times in South Australia and Western Australia. Each location included an early and late sowing treatment to generate variation in weather, particularly rainfall, daylength, and temperature. We evaluated the vigour NILs (Vig) in four environments in 2024, and the flowering-time NILs (FT), across eight environments in 2023 and 2024. Locations were Kapunda (–34.40, 138.86), Roseworthy (–34.53, 138.71), and Merredin (–31.49, 118.23). Early and late sowings were: Merredin 2023—26 May and 21 June; Merredin 2024—24 May and 20 June; Kapunda 2023—20 June and 21 July; and Roseworthy 2024—14 May and 28 June.

NILs were allocated to a randomized complete block design with three replicates. Plots comprised six 5-m rows with 25 cm row spacing and a target density of 50 plants m^− 2^. Seed were treated with P-Pickel T before to sowing. Crops were managed using locally recommended practices for nutrition, weed, pest, and disease control.

#### Measurements

Phenology was scored twice weekly to determine when 50% of plants in a plot had reached flowering, podding, and maturity. Phenological stages were expressed on a thermal time scale using a base temperature of 0 °C (Berger et al. 2006; Soltani et al. 2006). Canopy height was measured from the soil to the top of the canopy in the centre of the plot, and canopy cover was measured using NDVI (GreenSeeker, NTech Industries Inc., USA). At maturity, two lineal metres were harvested from the four central rows of each plot. Yield components including grain yield, seed number, and individual seed weight were determined.

Daily minimum and maximum temperature, rainfall, reference evapotranspiration, and solar radiation were obtained from the nearest Bureau of Meteorology station at each site.

#### Statistical analysis

Data were analysed separately for the CaLG3a (Vig) and CaLG3b (FT) NILs. ANOVA and linear mixed models were used to test for effects of genotype, environment, and their interaction on phenology, yield, and related traits (Genstat v20). Genotype (allele G vs. R) was modelled as a fixed effect, with environment, block, and genotype × environment as random terms. Pairwise contrasts between alleles were obtained using em means, with Holm-adjusted P-values. Environment-wise means and contrasts are presented with 95% confidence intervals. Model assumptions were verified by inspection of residual plots, and heterogeneity among environments was modelled with a varIdent structure when necessary.

## Results

### Experiment 1: developmental responses of NILs to photoperiod in controlled environment

#### Phenotypic variation

**Fig. 1:**
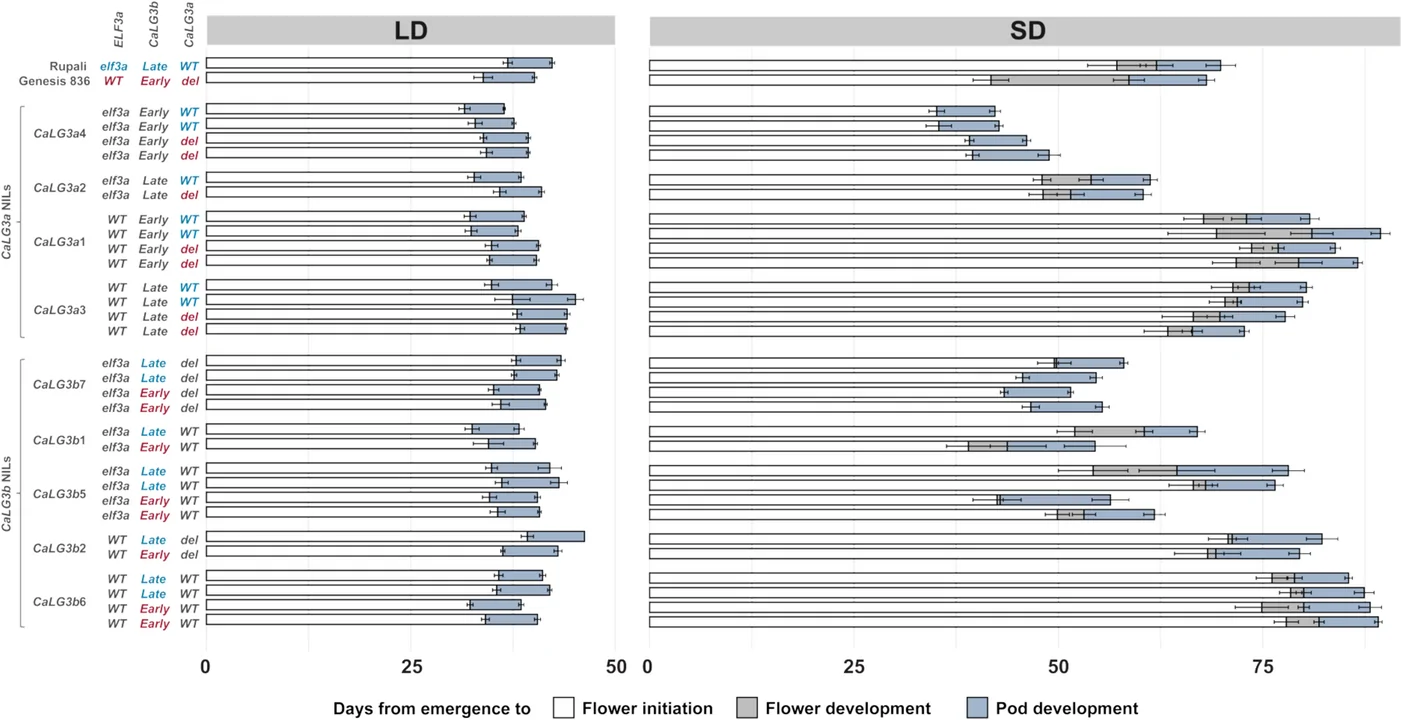
Days from seedling emergence to flower initiation (white bars), flower development (grey bars), and pod development (blue bars) in 30 chickpea Near-Isogenic Lines (NILs) and the parental accessions *Rupali* and *Genesis836* grown under long-day and short-day. Bars are mean ± standard error. Allelic composition at *ELF3a*, *CaLG3a*, and *CaLG3b* loci are indicated for each line

Phenotypic variation was observed among the NILs and parental genotypes for all traits, with marked differences between long- and short-day treatments (Fig. 1; Table 3, Table S3, Fig. S2). Under long day, reproductive transition resulted in successful flower development approximately 35 d after germination for all plants, progressing to pod development at approximately 6 d after DFD, with no or residual floral and pod abortions recorded (Table 3, Fig. S2). Under this condition, the parent cultivars were phenologically similar, with *Genesis836* showing a slightly but consistently earlier phenological timing (≈ 3 d) that was not translated into nodes. The NILs exhibited moderate variation, clustering in a narrow range of 29–56 days and 14–23 nodes between the onset of flowering and podding (Fig. 1, Fig. S2, Table 3).

Developmental progression was slower under non-inductive short day. Relative to long day, flower initiation, flower development and pod development were delayed by more than 20 d on average (DFI = 57.3 ± 15.5 d; DFD = 60.3 ± 16.9 d; DPD = 68.5 ± 16.3 d). This pattern was also evident for node number, with higher positions recorded for all traits under short day (Table 3; Fig. 1, Figs. S2-S3). The delay was also evident in the parental lines. While there were differences of less than 4 d and 3 nodes between *Genesis836* and *Rupali* for flower and pod development under short day, *Genesis836* initiated reproductive transition, on average, 15.3 d (7.6 nodes) earlier than *Rupali* (Table 3; Fig. 1 Fig. S3). Flower development was delayed in *Genesis836* for more than 17 d (6 nodes); these were the highest average floral abortions among all lines (Table S3). Such substantial delay indicates a strong daylength sensitivity for *Genesis836* and the presence of an “abortive” factor that blocks flower development under non-inductive photoperiods.

Differences between the NILs were accentuated by short day, with DFI, DFD and DPD varying by more than 60 days and 35 nodes (Table 3; Fig. 1, Table S3, Figs. S2-S3). The set of NILs also mirrored the contrasting response displayed by parental lines in their intervals, with some NILs sustaining extended abortive phases (over 30 days and 13 nodes), while others showed no developmental delay.

There was transgressive segregation in the NILs in both photoperiod regimes, confirming the presence of early and late alleles in both parental accessions. Extreme phenotypes were more commonly observed towards the late phenological spectrum under short day, where some NILs such as CaLG3b6 flowered more than 20 days and 10 nodes later than the parents (Table S3, Fig. S2).

Despite the photoperiod-dependent variation, correlations among phenological traits were strong and biologically consistent (Fig. S4). Flowering and podding traits, based on either days or nodes, were all highly correlated (*r* = 0.91–0.99), indicating a tightly coordinated progression of reproductive development that suggests common regulation. In contrast, weaker correlations were found between gap traits (*r* = 0.14–0.22) and between gaps and core phenological traits (*r* = 0.24–0.58), indicating that these are largely independent components rather than simple extensions of flowering time.

**Table 3:** Summary statistics of ten phenology traits recorded in 30 NILs derived from parent varieties *Rupali* and *Genesis836* grown under long or short day in a phytotron

Long day											
		DFI	DFD	DPD	NFI	NFD	NPD	DFDELAY^a^	DPDELAY	NFGAP^a^	NPGAP^a^
NILs	**Mean**	35.0	35.0	40.9	17.2	17.2	17.2	N/A	5.9	N/A	0.02 ± 0.1
	**SD**	2.7	2.7	3.3	1.7	1.7	1.8	N/A	1.4	N/A	0.1
	**Range**	29–47	29–47	33–56	14–22	14–22	14–23	N/A	3–17	N/A	0–2
	**Median**	35	35	41	17	17	17	N/A	6	N/A	0
Genesis836	**Mean**	33.8	33.8	40.1	15.2	15.2	15.2	N/A	6.2	N/A	N/A
	**SD**	3.0	3.0	3.5	0.4	0.4	0.4	N/A	0.7	N/A	N/A
	**Range**	30–40	30–40	35–47	15–16	15–16	15–16	N/A	5–7	N/A	N/A
	**Median**	33	33	40	15	15	15	N/A	6	N/A	N/A
*Rupali*	**Mean**	36.8	36.8	42.2	15.8	15.8	15.8	N/A	5.4	N/A	N/A
	**SD**	1.4	1.4	1.6	0.9	0.9	0.9	N/A	0.7	N/A	N/A
	**Range**	34–38	34–38	39–44	15–17	15–17	15–17	N/A	5–7	N/A	N/A
	**Median**	37	37	43	16	16	16	N/A	5	N/A	N/A
Short day											
		DFI	DFD	DPD	NFI	NFD	NPD	DFDELAY	DPDELAY	NFGAP	NPGAP
NILs	**Mean**	57.3	60.3	68.5	27.9	29.5	29.9	2.9	8.4	1.6	0.5
	**SD**	15.5	16.9	16.3	8.0	8.9	8.8	4.9	3.3	2.6	1.3
	**Range**	30–90	30–98	36–97	15–44	15–50	15–47	0–31	3–24	0–13	0–9
	**Median**	57	60	69	25	28	29	0	8	0	0
*Genesis836*	**Mean**	41.8	58.6	68.1	18.4	24.6	25.0	16.9	9.5	6.3	0.4
	**SD**	6.2	7.2	8.3	2.2	2.6	2.5	5.4	2.8	2.3	0.5
	**Range**	35–51	47–66	56–76	16–22	20–27	21–27	12–26	6–13	3–9	0–1
	**Median**	41	61.5	69.5	18	26	26	15.5	8.5	6	0
*Rupali*	**Mean**	57.1	62.0	69.9	26.0	27.3	28.6	4.9	7.9	1.3	1.3
	**SD**	9.4	8.4	5.2	4.1	3.6	3.2	5.3	4.8	1.5	2.6
	**Range**	48–76	48–76	66–81	23–34	23–34	24–34	0–14	4–18	0–4	0–7
	**Median**	55	61	69	24	27	28	5	7	1	0

^**a**^ N/A: No aborted flowers or pods recorded under long day

*DFI *days to flower initiation, *DFD* days to a fully developed flower, *DPD *days to a fully developed pod, *NFI *nodes to flower initiation, *NFD* nodes to a fully developed flower, *NPD *nodes to a fully developed pod

#### Allelic effects

**Fig. 2:**
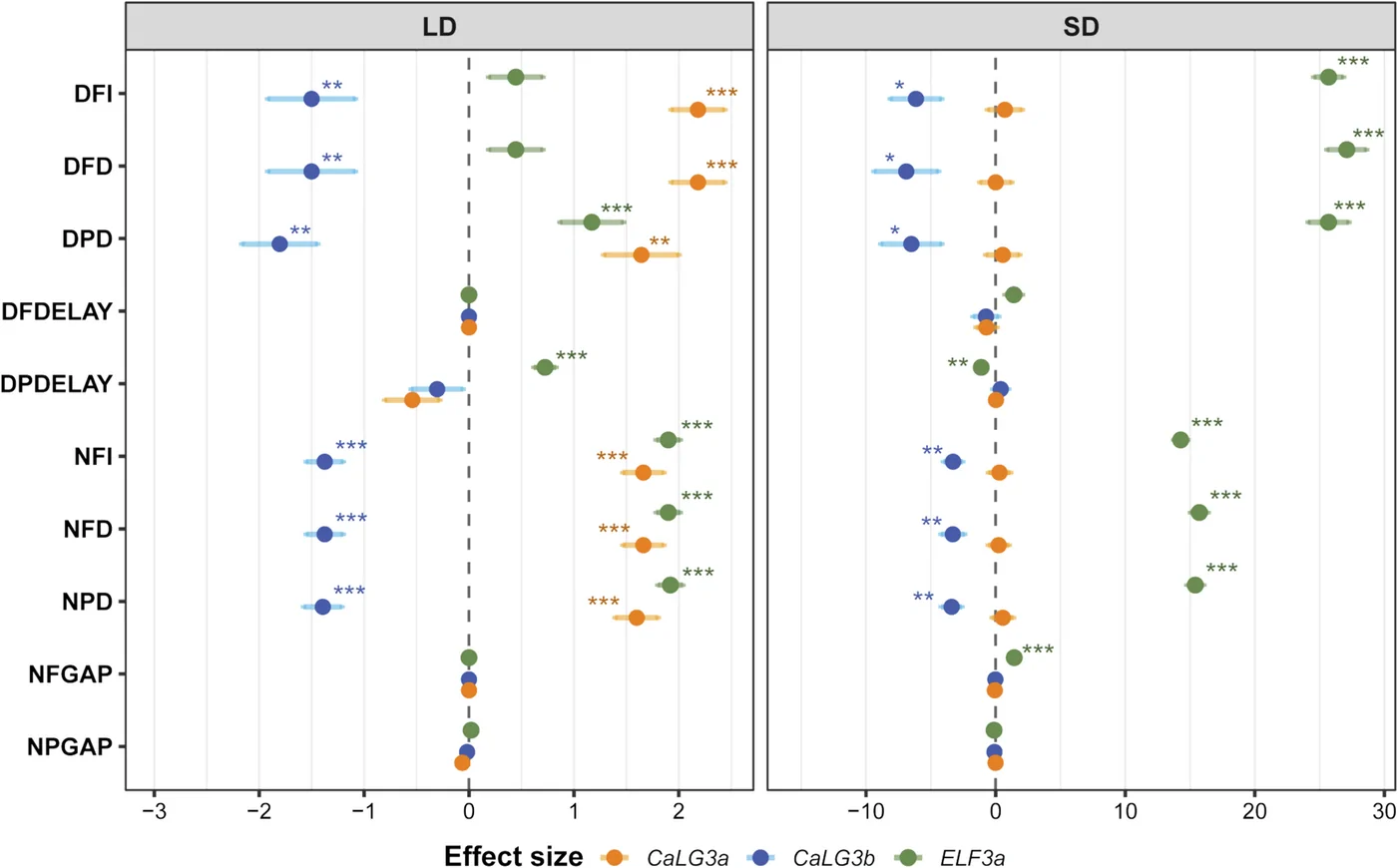
Allelic effect of *CaLG3a*, *CaLG3b* and *ELF3a* on ten phenological traits under long-day (LD) and short-day (SD). Each point represents the mean trait difference between NILs carrying the *Genesis836* allele and those carrying the *Rupali* allele (G-R contrast), and horizontal bars indicate ± standard error. Asterisks indicate statistical significance (*P < 0.05; **P < 0.01; ***P < 0.001) after FDR correction. Colours correspond to loci as indicated in the legend

Both *CaLG3a* and *CaLG3b* influenced phenology, with photoperiod × allele interactions detected for both loci (Fig. 2, Table S4). Pairwise contrasts between NILs segregating for *CaLG3b* revealed an earlier progression through all reproductive stages for lines carrying the *LG3b*^*Early*^ allele. The interaction is captured in a larger effect under short day, where this allele associated with an advancement of ≈ 6.1 d and 3.3 nodes, compared to 1.5 d and 1.3 nodes under long day. Similarly, for *CaLG3a* under long day, the *LG3a*^*Del*^ allele associated with delayed flowering and podding by approximately 2 d and 1 node. The phenological differences between NILs segregating for *CaLG3a* under short day were negligible. Irrespective of photoperiod, the number of aborted flowers or pods was unrelated to either *CaLG3a* or *CaLG3b*.

The effect of *ELF3a* was also assessed in lines differing for this locus but with the same genotype at *CaLG3a* and *CaLG3b*. Under long day, *ELF3a* showed a small but significant effect on flowering when measured as nodes, and in podding time. By contrast, under short day, *ELF3a* showed the largest and most consistent effect, with all three developmental traits delayed in NILs carrying the functional *ELF3a*^*WT*^ by more than 25 d and 14 nodes (Fig. 2, Table S4). Consequently, photoperiod sensitivity, determined as the difference in phenology between plants grown under short day and long day, was greatly reduced in all genotypic combinations containing the *elf3a* allele, particularly when combined with *LG3b*^*Early*^ (Fig. S5).

*ELF3a* was also the only locus associated with flower and pod abortions, which were less common in NIL families carrying the *elf3a* allele (Figs. 1 and 2, Fig. S3, Table S3). However, the magnitude of its effect was not enough to explain the marked differences between the parental lines in their abortion phenotype.

**Fig. 3:**
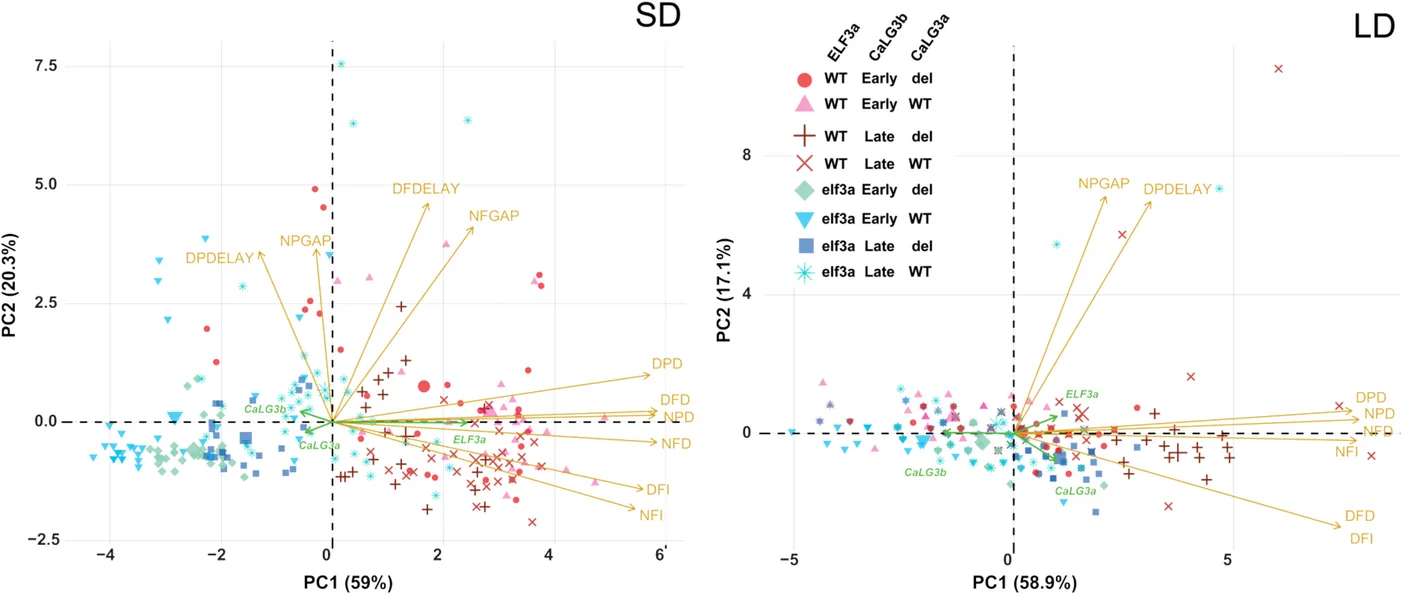
Principal component analysis of phenological traits for chickpea near-isogenic lines (NILs) under short- (SD) and long day (LD). Points represent individual plants, coloured and shaped by their allelic combination at *ELF3a*, *CaLG3b*, and *CaLG3a* loci as indicated in the legend. Trait loadings (golden arrows) indicate the contribution of phenology traits to the first two principal components, while genotype loadings (green arrows) show the influence of allelic variation on the response

Principal Component Analysis aligns with a strong statistical interaction between genotype and environment (Fig. 3). The loading structure was consistent in both photoperiods, with PC1 primarily driven by developmental traits while PC2 captured differences in the length of the intervals between them. Allelic composition correlated with PC axes, but with distinct patterns between environments. Under long day, *CaLG3b* aligned with PC1, indicating that this locus was the primary contributor to phenology under inductive photoperiods. Allelic effects at *CaLG3a* and *ELF3a* were directionally aligned but exhibited smaller correlations. In contrast, under short day, *ELF3a* had a dominant effect on PC1, with a smaller but detectable contribution of *CaLG3b*. NIL groups clustered accordingly: lines carrying multiple late or early alleles clustered toward high and low PC1 values, respectively, although the magnitude of their differences/displacement varied with daylength.

#### Interactions between loci

We observed a strong interaction between the three loci. Under short day, NILs with either a functional *ELF3a*^*WT*^ allele or bearing the deletion in the *FT* region (*LG3a*^*Del*^) showed little difference in phenology regardless of their genotype at *CaLG3b* (e.g. CaLG3b2, CaLG3b6 and CaLG3b7). By contrast, in NILs with an *elf3a*/*LG3a*^*WT*^ background (CaLG3b1 and CaLG3b5), *LG3b*^*Early*^ plants flowered, on average, more than 16 d and 7 nodes earlier than *LG3b*^*Late*^ plants (Figs. S6-S7, Table S3).

Similarly, no significant differences were detected in CaLG3a NILs carrying either an *ELF3a*^*WT*^ or *LG3b*^*Late*^ allele, while in an *elf3a*/*LG3b*^*Early*^ background, *LG3a*^*WT*^ segregants showed earlier flowering compared to *LG3a*^*Del*^ segregants (Figs. S6-S7, Table S3). The effect of *CaLG3a* was weaker than that of *CaLG3b*, with differences of ≈ 4.5 d and 3 nodes for flower initiation and development.

Collectively, these findings indicate that, under short photoperiod, *CaLG3b* and *CaLG3a* interacted synergistically to promote flowering, whereas *ELF3a* acted as a suppressor of this interaction, exerting an epistatic effect over the *LG3b*^*Early*^/*LG3a*^*WT*^ combination. When combined, the photoperiod-dependent effect of these loci and their interactions resulted in a broad phenotypic spectrum determined by allelic composition (Fig. S8).

### Experiment 2: environmental conditions in the field

**Fig. 4:**
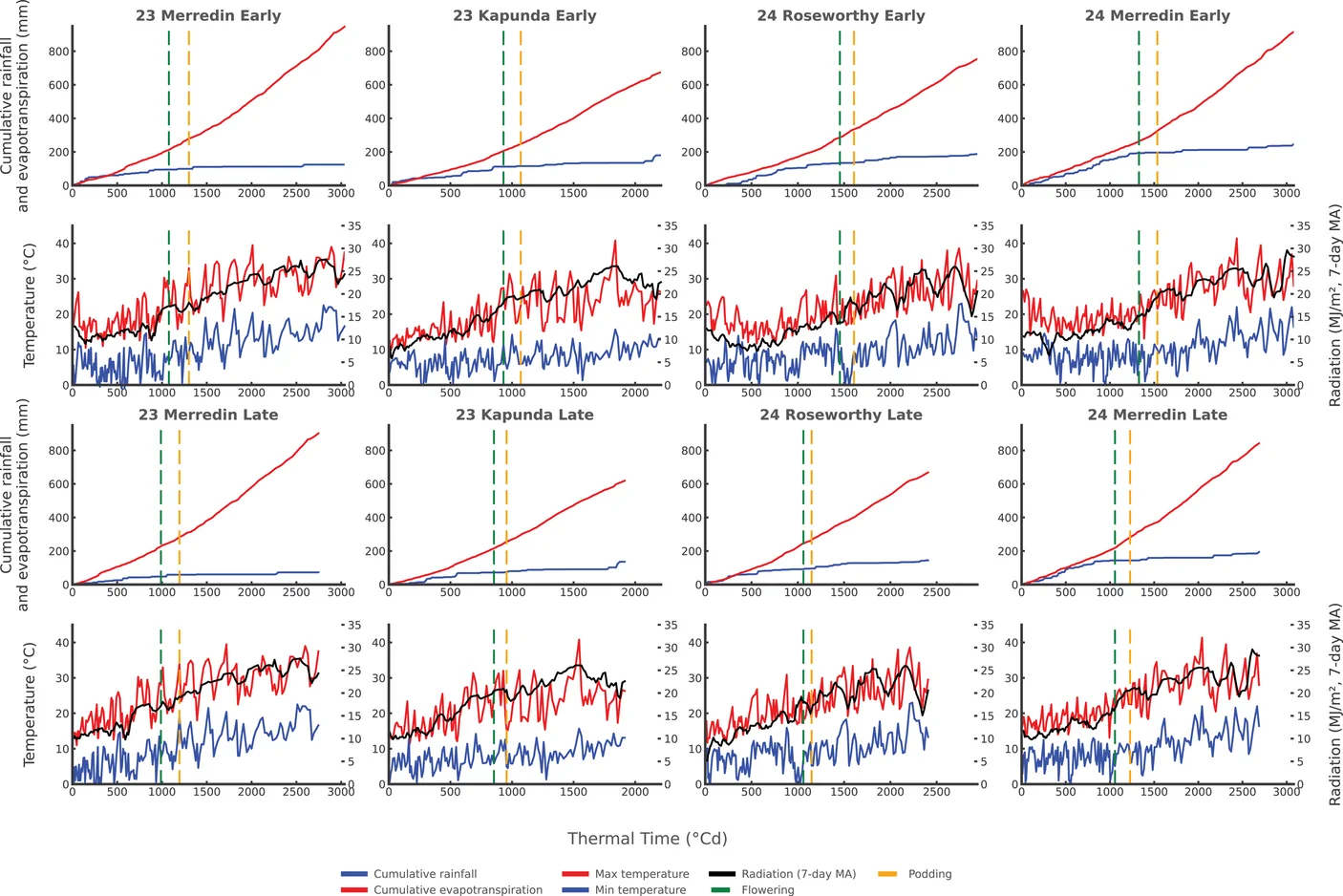
Seasonal growing conditions for eight environments resulting from the combination of locations, seasons and sowing dates (early, late): Merredin (2023, 2024), Kapunda (2023), and Roseworthy (2024). For each environment, the upper panel shows cumulative rainfall (blue) and evapotranspiration (red), and the lower panel shows daily maximum (red) and minimum (blue) temperature together with incident radiation expressed as a 7-day moving average (black, right axis). Vertical dashed lines mark flowering (green) and podding (orange). The x-axis is thermal time (base temperature = 0 °C) from sowing until 1 December

Growing conditions varied among the eight environments (Fig. 4). Cumulative evapotranspiration consistently exceeded cumulative rainfall. By 1 December, evapotranspiration approached 900–950 mm at Merredin and ~ 700–800 mm at Roseworthy and Kapunda, whereas cumulative rainfall was generally at or below 200 mm.

Across all sites, the flowering to podding period was short (~ 100–200°Cd, Fig. 4). Early sowings flowered under cooler temperatures and lower evapotranspiration, with podding generally occurring before the onset of extreme heat (> 30 °C). In contrast, later sowings delayed reproductive development, resulting in exposure to maximum temperatures that frequently exceeded 30 °C during reproduction. Averaged across sites, early sowings experienced 6.0 d above 25 °C, 2.3 d above 28 °C, and 1.0 d above 30 °C from flowering until 300°Cd post-flowering. In contrast, late sowings had 6.5, 3.8, and 1.5 d above the same thresholds.

#### Yield, phenology and growth – CaLG3a NILs

**Fig. 5:**
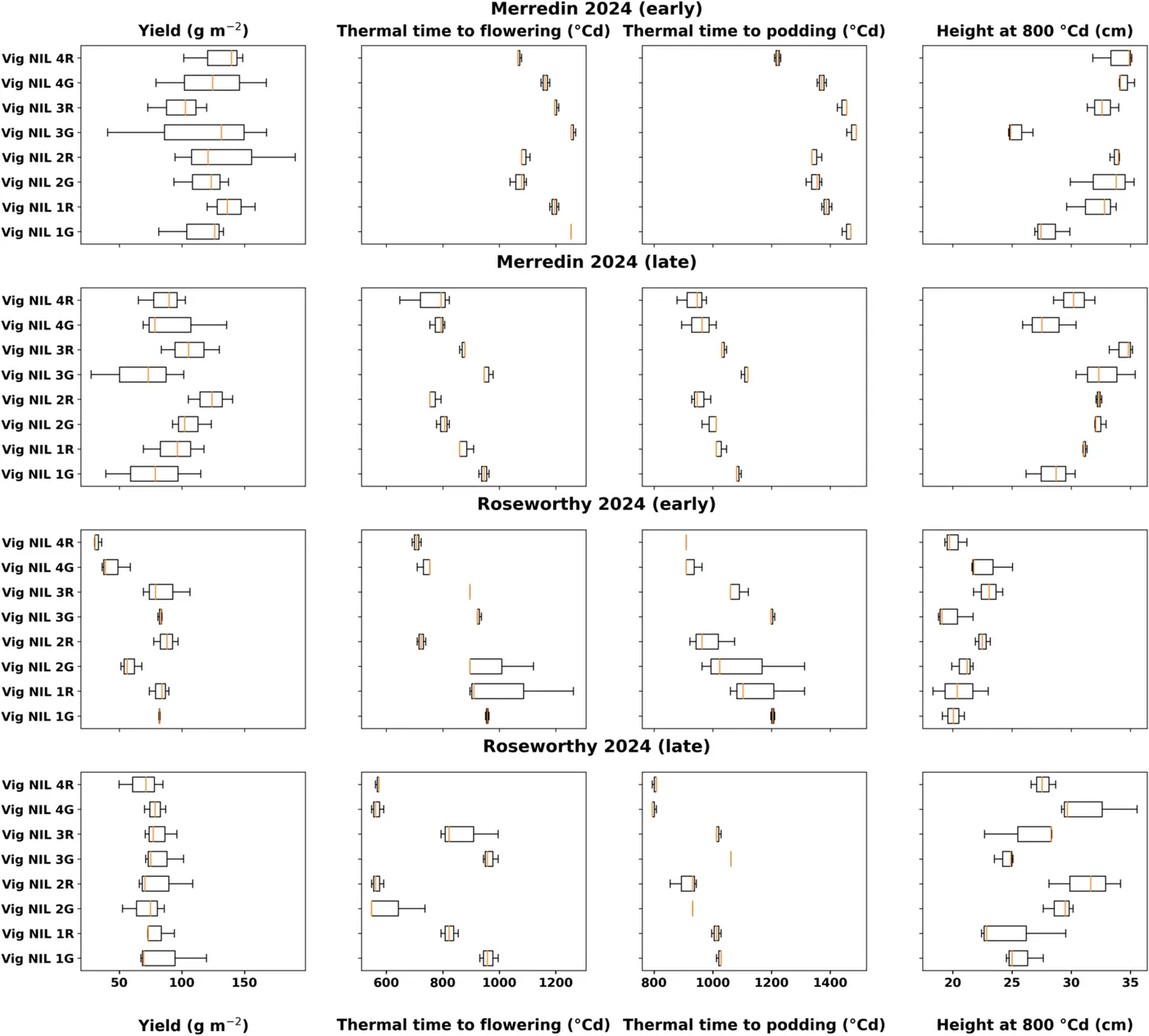
Grain yield, thermal time from emergence to flowering and podding, and plant height at 800°Cd for CaLG3a (Vig) NILs across environments. Each panel shows the distribution of NIL pair members (G and R) within environments. The central boxplot line represents the median, the box bounds indicate the 25th and 75th percentiles, and the whiskers extend to 1.5 × the interquartile range. Outliers beyond the whiskers are plotted individually as points

Yield of the CaLG3a (Vig) NILs varied across environments (*p* < 0.001, Fig. 5; Table 4). The highest yielding environment was for early-sown crops at Merredin 2024 (mean of genotypes: 121 ± 7 g m⁻²) and the lowest was for early-sown crops at Roseworthy 2024 (69 ± 4.6 g m⁻²). For early-sown crops at Merredin 2024, the top-yielding Vig lines were Vig NIL 1R (138 g m⁻²) and Vig NIL 2R (135 g m⁻²). Across environments, Vig NIL mean yields ranged from 79 g m⁻² in Vig NIL 4R to 107 g m⁻² in Vig NIL 2R (Table 5). Pairwise differences ranged from 6.2 g m⁻² (Vig 3G vs. 3R) to 18.5 g m⁻² (Vig 2G vs. 2R).

**Table 4:** Mean ± standard error of yield and phenological traits across environments for CaLG3b (FT) and CaLG3a (Vig) NILs

Environment	NIL class	Yield (g m^−^²)	Thermal time to flowering (°Cd)	Thermal time to podding (°Cd)	Flowering-podding interval (°Cd)
Kapunda 2023 early sowing	FT	255 ± 7.9	835 ± 14.8	943 ± 12.5	108 ± 3.4
Kapunda 2023 late sowing	FT	160 ± 5.6	714 ± 16.0	795 ± 12.7	78 ± 5.0
Merredin 2023 early sowing	FT	38 ± 2.7	981 ± 20.7	1205 ± 28.7	224 ± 13.0
Merredin 2023 late sowing	FT	30 ± 2.3	916 ± 26.0	1063 ± 22.0	147 ± 17.6
Merredin 2024 early sowing	FT	117 ± 6.4	1195 ± 15.7	1415 ± 9.9	220 ± 7.6
Merredin 2024 late sowing	FT	70 ± 5.1	915 ± 15.1	1071 ± 13.9	156 ± 6.1
Roseworthy 2024 early sowing	FT	98 ± 5.7	957 ± 20.6	1141 ± 22.3	183 ± 19.6
Roseworthy 2024 late sowing	FT	102 ± 4.8	872 ± 28.9	1010 ± 14.8	137 ± 18.7
Merredin 2024 early sowing	Vig	121 ± 7.0	1163 ± 15.5	1382 ± 16.4	219 ± 8.4
Merredin 2024 late sowing	Vig	94 ± 5.9	845 ± 16.9	1013 ± 13.7	167 ± 5.6
Roseworthy 2024 early sowing	Vig	69 ± 4.6	871 ± 30.1	1072 ± 28.3	201 ± 14.6
Roseworthy 2024 late sowing	Vig	79 ± 3.5	742 ± 36.7	945 ± 20.1	203 ± 22.0

**Table 5:** Mean ± standard error of yield, its components and phenological traits for near-isogenic lines (NILs) averaged across eight rainfed environments

NIL	Yield (g m^−^²)	100-grain weight (g)	Seed number (m²)	Biomass (g m^−^²)	Harvest index	Thermal time to flowering (°Cd)	Thermal time to podding (°Cd)	Flowering-podding interval (°Cd)
FT NIL 1G	110 ± 13.7	21 ± 0.5	505 ± 60.6	210 ± 43.8	0.34 ± 0.02	788 ± 30.2	945 ± 36.8	157 ± 11.4
FT NIL 1R	115 ± 14.3	22 ± 0.4	513 ± 63.0	232 ± 49.0	0.36 ± 0.03	784 ± 36.7	975 ± 41.2	191 ± 19.7
FT NIL 2G	122 ± 17.0	21 ± 0.5	572 ± 79.8	212 ± 42.1	0.37 ± 0.03	932 ± 26.6	1106 ± 36.3	173 ± 17.2
FT NIL 2R	109 ± 15.4	22 ± 0.4	496 ± 70.2	234 ± 52.6	0.32 ± 0.03	975 ± 29.8	1141 ± 38.0	165 ± 13.6
FT NIL 3G	91 ± 13.5	19 ± 0.3	472 ± 72.2	203 ± 44.2	0.28 ± 0.03	945 ± 22.5	1100 ± 35.8	155 ± 19.3
FT NIL 3R	89 ± 13.2	20 ± 0.3	443 ± 66.0	204 ± 43.8	0.26 ± 0.02	990 ± 25.8	1132 ± 35.5	142 ± 13.9
FT NIL 4G	120 ± 20.2	20 ± 0.2	590 ± 99.0	202 ± 41.7	0.37 ± 0.02	960 ± 37.8	1091 ± 48.5	131 ± 13.3
FT NIL 4R	115 ± 14.1	22 ± 0.4	524 ± 66.4	231 ± 53.7	0.30 ± 0.03	1005 ± 24.9	1146 ± 30.8	136 ± 15.2
Vig NIL 1G	90 ± 8.8	16 ± 0.6	572 ± 66.3	315 ± 47.2	0.30 ± 0.02	1037 ± 42.5	1192 ± 55.2	156 ± 22.2
Vig NIL 1R	99 ± 8.2	16 ± 0.5	617 ± 67.2	392 ± 48.2	0.30 ± 0.01	979 ± 50.7	1145 ± 48.6	166 ± 13.6
Vig NIL 2G	89 ± 8.4	25 ± 0.5	364 ± 38.3	369 ± 49.1	0.32 ± 0.03	864 ± 56.9	1094 ± 53.3	230 ± 27.2
Vig NIL 2R	107 ± 9.9	25 ± 0.3	434 ± 42.6	407 ± 52.6	0.33 ± 0.03	787 ± 57.4	1050 ± 53.9	263 ± 20.5
Vig NIL 3G	87 ± 10.7	23 ± 0.4	376 ± 48.8	411 ± 84.8	0.23 ± 0.05	1028 ± 40.9	1213 ± 48.5	186 ± 20.7
Vig NIL 3R	93 ± 5.9	24 ± 0.5	397 ± 28.9	370 ± 28.1	0.28 ± 0.02	960 ± 44.1	1145 ± 52.9	185 ± 17.3
Vig NIL 4G	86 ± 12.3	21 ± 0.5	422 ± 63.2	297 ± 42.0	0.37 ± 0.02	813 ± 65.8	1013 ± 65.3	200 ± 10.0
Vig NIL 4R	79 ± 11.5	20 ± 0.4	400 ± 58.3	348 ± 51.2	0.33 ± 0.03	782 ± 61.5	972 ± 51.2	190 ± 12.0

Phenology of CaLG3a (Vig) NILs varied across environments (*p* < 0.001, Fig. 5). Flowering was earliest in late-sown crops at Kapunda 2023 (714°Cd) and latest for early-sown crops at Merredin 2024 (1179°Cd). Across NILs, thermal time from emergence to flowering ranged from 782°Cd in Vig NIL 4R (carrying all three early flowering alleles: *elf3a/LG3b*^*Early*^*/LG3a*^*WT*^) to 1037°Cd in Vig NIL 1G (*ELF3a*^*WT*^*/LG3b*^*Early*^*/LG3a*^*Del*^). Differences within Vig NIL pairs in flowering time spanned from approx. 40°Cd to over 80°Cd. The flowering–podding interval of Vig NILs varied with environment (*p* < 0.001). The shortest intervals were observed for late-sown crops at Merredin 2024 (167°Cd), and the longest was for early-sown crops at Merredin 2024 (219°Cd). Across NILs, means ranged from 156°Cd in Vig NIL 1G (*ELF3a*^*WT*^*/LG3b*^*Early*^*/LG3a*^*Del*^) to 264°Cd in Vig NIL 2R (*elf3a/LG3b*^*Late*^*/LG3a*^*WT*^). The flowering-podding interval tended to be greater in NILs carrying the early *elf3a* allele. Within-pair contrasts typically spanned 15–30°Cd and varied with the interaction between environment and NIL (*p* < 0.001).

Plant height at 800°Cd after emergence varied with environment (*p* < 0.001), from 21.3 cm for early-sown crops at Roseworthy 2024 (21.3 cm) to 31.7 cm for early-sown crops at Merredin 2024, the environment with the slowest phenological development. Across NILs, mean height ranged from 25.6 cm in Vig NIL 3G (*ELF3a*^*WT*^*/LG3b*^*Late*^*/LG3a*^*Del*^) to 30.0 cm in Vig NIL 2R (*elf3a/LG3b*^*Late*^*/LG3a*^*WT*^). Differences within Vig NIL pairs typically spanned 2–5 cm. While the Rupali allele at *LG3a* (*LG3a*^*WT*^) was often the taller member of each NIL pair, some pairs expressed contrasting height responses between environments. The environment × NIL interaction (*p* < 0.001) was reflected in inconsistent height rankings of Vig NILs across sites (Fig. 5).

#### Yield, phenology and growth – CaLG3b NILs

**Fig. 6:**
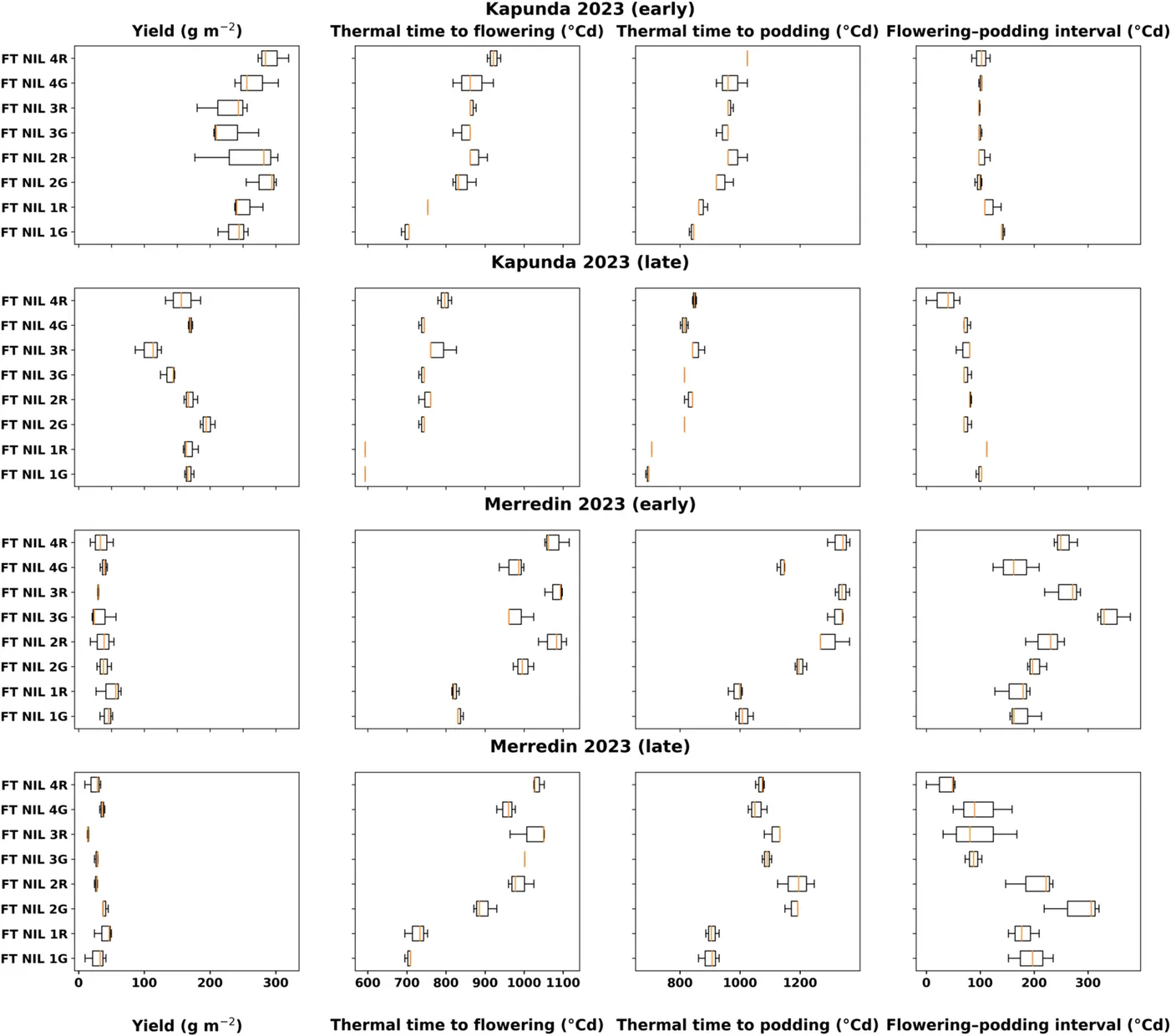
Grain yield, thermal time from emergence to flowering, thermal time to podding, and flowering–podding interval for CaLG3b (FT) NILs across 2023 environments. Each panel shows the distribution of NIL pair members (G and R) within environments. The central boxplot line represents the median, the box bounds indicate the 25th and 75th percentiles, and the whiskers extend to 1.5 × the interquartile range. Outliers beyond the whiskers are plotted individually as points

**Fig. 7:**
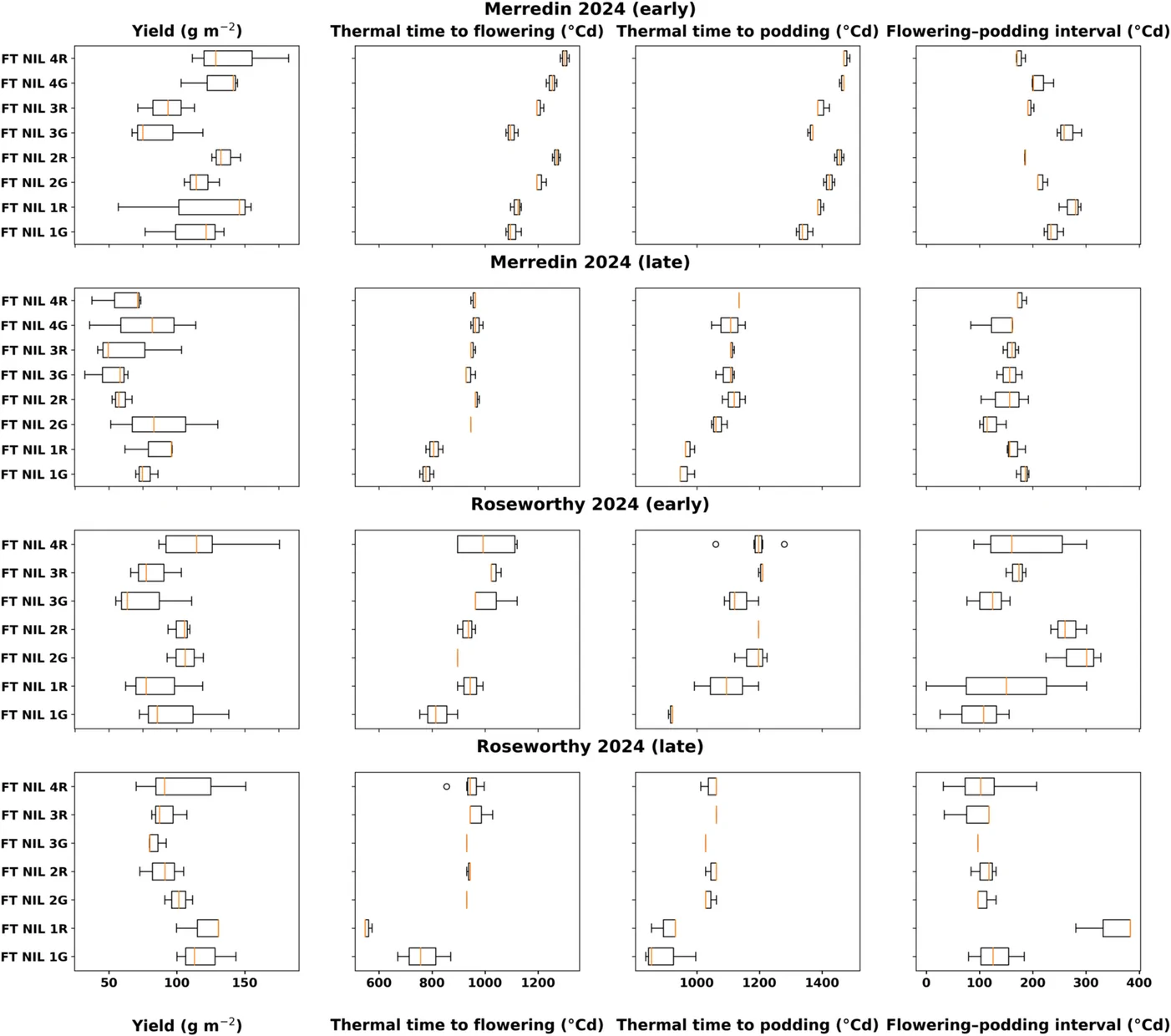
Grain yield, thermal time from emergence to flowering, thermal time to podding, and flowering–podding interval for CaLG3b (FT) NILs across 2024 environments. Each panel shows the distribution of NIL pair members (G and R) within environments. The central boxplot line represents the median, the box bounds indicate the 25th and 75th percentiles, and the whiskers extend to 1.5 × the interquartile range. Outliers beyond the whiskers are plotted individually as points

Yield of CaLG3b (FT) NILs varied with environment (*p* < 0.001, Figs. 6 and 7), in a range from 30 ± 2.3 g m⁻²) for late-sown crops at Merredin 2023 to 255 ± 7.9 g m⁻² for early-sown crops at Kapunda 2023. For early-sown crops at Kapunda 2023, the highest yielding FT line was FT NIL 4R (*LG3b*^*Late*^; 292 g m⁻²), followed by FT NIL 2G (*LG3b*^*Early*^; 283 g m⁻²). Across environments, FT NIL means ranged from 89 g m⁻² in FT NIL 3R to 122 g m⁻² in FT NIL 2G (Table 5), with within-pair contrasts from 2.1 g m⁻² (FT 3G vs. 3R) to 12.9 g m⁻² (FT 2G vs. 2R).

Phenology of FT NILs also varied with environment (*p* < 0.001, Figs. 6 and 7), from 714°Cd in late-sown crops at Kapunda in 2023 to 1179°Cd in early-sown crops at Merredin in 2024. Within the FT set, flowering time ranged from 784°Cd in FT NIL 1R (*elf3a/LG3b*^*Late*^*/LG3a*^*Del*^*)* to 1005°Cd in FT NIL 4R (*ELF3a*^*WT*^*/LG3b*^*Late*^*/LG3a*^*Del*^*)*. FT NIL pair 1, the only pair in this set carrying *elf3a*, was consistently earlier to flower and pod across environments.

The flowering–podding interval of FT NILs varied with environment (*p* < 0.001), in a range from 78°Cd at Kapunda 2023 late sowing to 224°Cd at Merredin in 2023 early sowing. Across NILs, mean flowering-podding interval ranged from 132°Cd in FT NIL 4G (*ELF3a*^*WT*^*/LG3b*^*Early*^*/LG3a*^*Del*^*)* to 191°Cd in FT NIL 1R (*elf3a/LG3b*^*Late*^*/LG3a*^*Del*^*)*. Within-pair contrasts were generally moderate, with a strong environment × NIL interaction (*p* < 0.001).

#### NIL pair comparisons

**Fig. 8:**
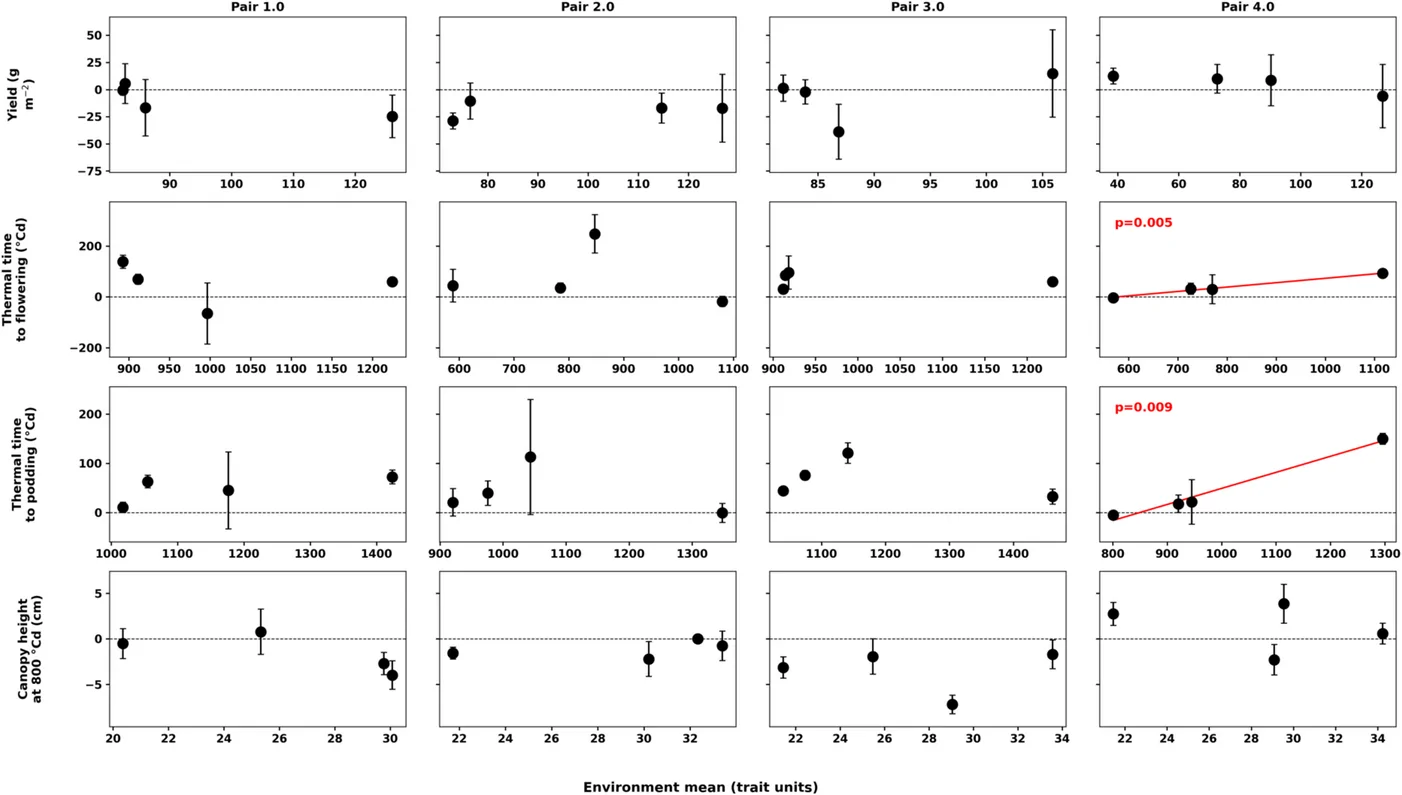
Allelic differences (NILG − NILR) in the expression of selected crop traits—grain yield, thermal time to flowering, thermal time to podding, and canopy height at 800°Cd—for CaLG3a vigour (Vig) near-isogenic line (NIL) pairs across environments. Each column represents a NIL pair, and each point represents an environment, plotted against the environment mean for the corresponding trait. Error bars indicate ± 1 standard error of the allelic difference. Solid red lines denote significant linear regressions (*p* < 0.05), fitted within the observed data range only; non-significant relationships are shown without regression lines. The dashed horizontal line indicates no difference between alleles. Y-axis limits are standardised within each trait to allow direct comparison among NIL pairs

Figure 8 examines differences in trait expression between CaLG3a (Vig) near-isogenic lines (NILs) carrying alternative alleles (G − R) as a function of the environmental mean for each trait, with the dashed line representing the null hypothesis of no difference. Differences in grain yield between NILs were small and varied inconsistently with environmental mean yield (Fig. 8), with all four NIL pairs showing non-significant relationships (*p* > 0.05).

In contrast, clear allelic effects were detected for phenological development. For both flowering and podding time, plants carrying the G allele of Vig NIL3 and Vig NIL4 reached flowering and podding later than those carrying the R allele across environments (*p* < 0.001). For Vig NIL4, the magnitude of this difference increased with the environmental mean of the trait, resulting in a significant relationship between allelic difference and environment mean for both flowering time (*p* = 0.005) and podding time (*p* = 0.009). The remaining Vig NIL pairs displayed flat or inconsistent responses to the environmental mean for both traits (*p* > 0.10).

**Fig. 9:**
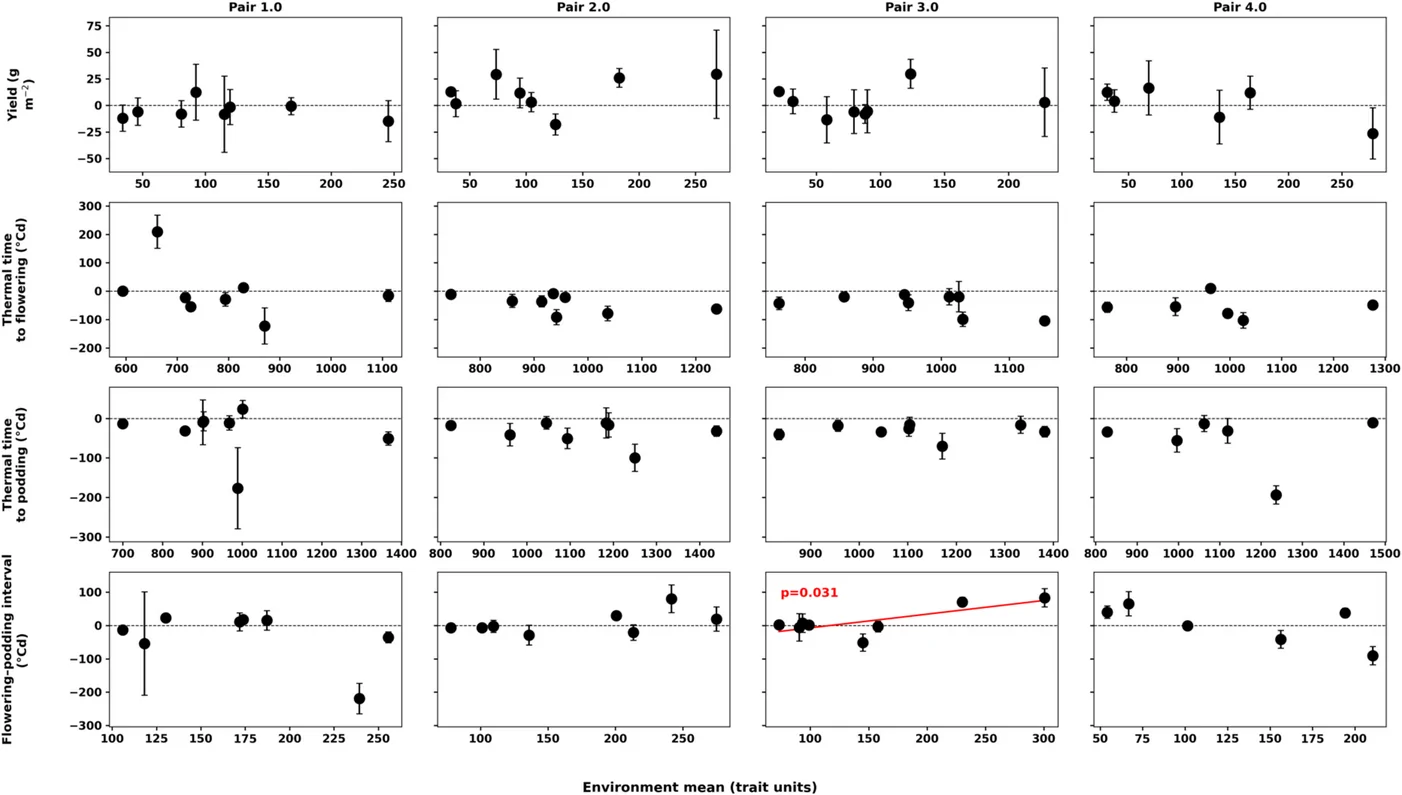
Allelic differences (NILG − NILR) in the expression of selected crop traits—grain yield, thermal time to flowering, thermal time to podding, and the flowering–podding interval—for flowering-time (FT) near-isogenic line (NIL) pairs across environments. Each column corresponds to a NIL pair, with points representing individual environments plotted against the environment mean for the respective trait. Error bars show ± 1 standard error of the allelic difference. Significant linear regressions (*p* < 0.05) are indicated by solid red lines and are constrained to the observed data range; no regression line is shown where relationships are non-significant. The dashed horizontal line represents no difference between alleles. Y-axis scales are standardised within each trait to facilitate comparison across NIL pairs

Canopy height at 800°Cd showed no evidence of allelic divergence, with differences between NILs typically < 3 cm across environments (*p* > 0.10). Across environments, yield differences between CaLG3b (FT) NILG and NILR were generally small (Fig. 9). NIL pairs 1–3 showed no yield differences (*p* ≥ 0.41). In contrast, NIL pair 4 exhibited a significant genotype × environment interaction (*p* = 0.03), characterised by an increasingly negative NILG − NILR yield difference with increasing environment mean yield. Although allelic differences crossed zero in some environments, the fitted regression had a significant negative slope, indicating that yield penalties for NILG tended to increase in higher-yielding environments.

## Discussion

Genes with strong flowering effects are typically discovered first because of their clear phenotypic impact, and often determine broad adaptation groups within crops (e.g. sowing window, season length, latitude). Such genes, like *Ppd*/*PHYA* in common bean or *ku*/*FTc* locus in lupin, provide coarse adjustment of phenology and have been crucial for the domestication and/or adaptation of these crops (Wallace et al. 1993; Weller et al. 2019; Gladstones and Hill 1969; Nelson et al. 2017). However, crops encounter large local environmental heterogeneity, and breeders increasingly seek to exploit minor-effect loci and harness their interactions with major regulators to fine-tune phenotype and environment.

Our NIL comparisons illustrate this principle, showing that chickpea phenology emerges from additive and interactive effects among *ELF3a*,* CaLG3b* and *CaLG3a*. Consistent with previous studies, the relative effects ranked *ELF3a* > *CaLG3b* > *CaLG3a*/*FT cluster* (Atieno et al. 2021). Across both long- and short-days in a controlled environment, the interactions between these loci resulted in a broad spectrum of flowering phenotypes. Notably, we show that similar flowering times can be achieved through different allelic combinations that can differ markedly for other phenological components. For instance, despite comparable times for first open flower, *Genesis836* displayed substantially higher floral abortion rates under short days than *Rupali*, highlighting potential trade-offs in contrasting environments.

### Model for CaLG3a, CaLG3b and ELF3a interaction

The functional roles and interactions of these loci align with findings in other species. *FT* homologues act as floral promoters across plant taxa, including legumes (Luo et al. 2022; Weller and Ortega 2015). In chickpea, a large deletion removing *FTa2* and part of the *FTa1-FTa2* intergenic region in the *FTa1*-*FTa2*-*FTc* cluster was previously proposed to accelerate flowering (Ortega et al. 2019), whereas in this study the deletion was associated with later phenology. This discrepancy likely reflects the complex regulation of *FT* genes involving multiple cis-regulatory elements and trans-acting regulators (Zhou et al. 2026; Gao et al. 2026). Since the *FTa1*-*FTa2* intergenic region is known to influence *FT* expression in other temperate legumes (Jaudal et al. 2013; Rajandran et al. 2022), the phenotype associated with this allele may depend on the background-specific availability of factors that interact with this region.

**Fig. 10:**
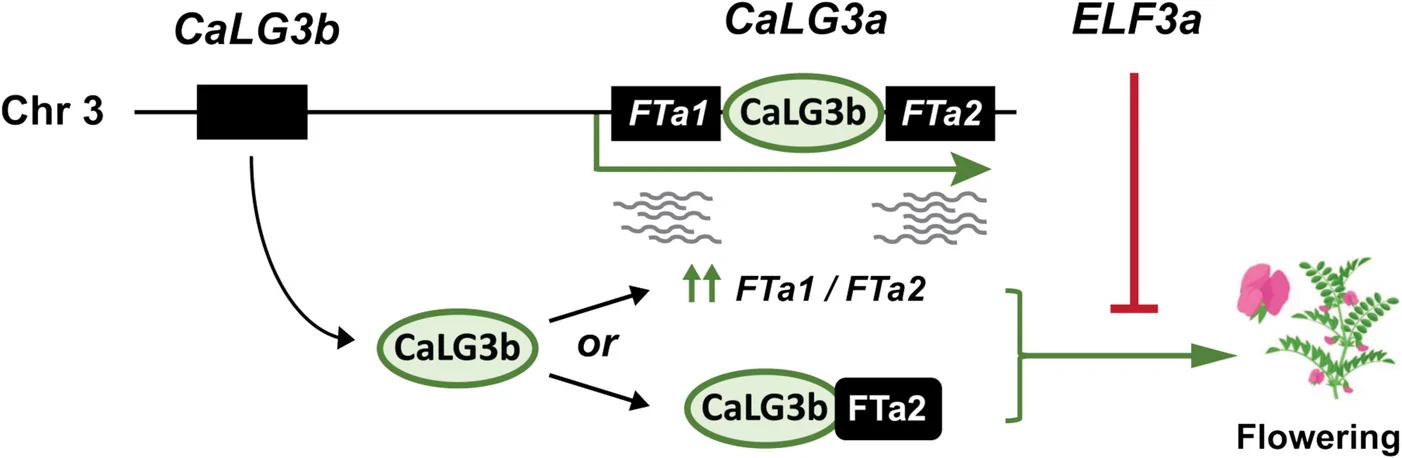
Hypothetic model of interaction between *CaLG3b*, *CaLG3a* and *ELF3a*, based on the phenotypes of the NILs characterized in this study. *CaLG3b* acts as a floral promoter that can either bind the *FTa1*-*FTa2* intergenic region to upregulate these genes, interact with FTa2 protein to promote flowering, or both. *ELF3a* acts as a potent floral suppressor, epistatic to *CaLG3a* and *CaLG3b*

By contrast, legume *ELF3* orthologues are strong floral repressors within the evening complex alongside *ELF4* and *LUX*, acting upstream of *FT* and other floral promoters to delay flowering under non-inductive conditions (Ridge et al. 2017; Weller et al. 2012; Yue et al. 2017). While the molecular identity and function of *CaLG3b* remain unresolved, our results support a model in which *CaLG3b* promotes flowering potentially through interaction with FTa2 protein or binding to regulatory elements within the intergenic region, but only in the absence of *ELF3a*-mediated repression (Fig. 10). While such interactions remain hypothetical and require functional validation, this model explains both the additive behaviour of the loci and the intermediate phenotypes observed in NILs carrying mixed allele combinations, reinforcing the idea that chickpea phenology, like in many other species, emerges from a network of interacting promoters and repressors (Zhao et al. 2025).

### In-field test of allelic effect

Although controlled environments allow to isolate genetic factors, they usually do not capture the complexity of the field, where daily and seasonal variation in temperature, daylength, radiation and soil water availability impact the phenotype (Aphalo and Sadras 2021; Blanchy et al. 2025). Accordingly, phenological responses may not scale from simplified platforms to full field trials (Lake et al. 2025). In the present study, allelic effects detected under controlled conditions were also evident in the field, particularly for NILs differing at *ELF3a*, although their magnitude was reduced. However, even small shifts in phenology may have adaptive implications under Australian dryland conditions, where delayed podding increases the risk of exposure to terminal drought and heat, whereas accelerated development can enhance stress escape at the expense of reduced biomass and frost risk. The NIL framework is especially informative in this context, attributing these modest phenology shifts to defined genomic regions while controlling for genetic background noise.

### Agronomical implications

Any trait or trait-related allele can be adaptive depending on the environmental scenario (Tardieu 2011; Siddiq et al. 2024). Across the eight field environments assessed, NILs varied more for yield than for phenology. While both sets of NILs (*LG3a*/Vig and *LG3b*/FT) expressed allelic differences in flowering and podding time, these rarely correlated with yield. Most NIL pairs were neutral for yield across sites and seasons, highlighting the strong environmental buffering of yield (Berger et al. 2006). Only FT NIL pair 4 showed a consistent yield penalty associated with one allele in environments above 255 g m^− 2^. Such context-dependent effects align with previous reports that yield benefits from single-locus substitutions are rarely robust across environments (Sadras and Richards 2014). Instead, the primary value of these loci in breeding lies in targeted phenological adjustment—e.g., avoiding late-season heat or drought—rather than direct yield enhancement.

Our work also offers some insight into the genetic control of floral abortions, a major constraint for chickpea productivity (Berger et al. 2004). Chickpea reproductive success is typically limited by a syndrome of low temperature and radiation, and high humidity (Gimenez et al. 2024). However, we observed marked differences in reproductive progression between long- and short-day in our phytotron experiment, despite similar temperature and radiation. Genotypes carrying a functional *ELF3a* allele exhibited delayed or aborted flower development under short day, suggesting that daylength itself may limit floral transition in some genetic backgrounds. Similar adaptive responses occur in other crops including wheat and soybean where photoperiod signalling the end of the growing season accelerates development (Ghiglione et al. 2008; Nico et al. 2015). These findings suggest that photoperiod modulation of reproductive development may be more widespread in chickpea than currently recognised, playing an adaptive role on this trait alongside soil moisture (Li et al. 2022).

These loci may also affect plant architecture, an important breeding target due to its effect on harvestability and yield (Erskine et al. 1988; Siddique et al. 2013; Silva-Perez et al. 2022; Hilioti et al. 2024). In the Vig NILs, canopy architecture diverged consistently across environments, with *CaLG3a*^*Del*^ genotypes generally shorter and less vigorous than their *CaLG3a*^*WT*^ counterparts. While reduced height can limit light interception and biomass accumulation, it may be advantageous by lowering lodging risk, illustrating the broader concept of trade-offs between vigour and stress adaptation. The co-localisation of flowering and vigour QTL at the *FT* gene cluster (Ortega et al. 2019) suggests pleiotropy or tight linkage, indicating that allelic selection for phenology cannot be considered independently of canopy traits.

Taken together, our findings emphasise both the opportunities and the limitations of manipulating flowering loci in chickpea. The capacity to combine major (*ELF3a*) and intermediate (*CaLG3b*, *FT* cluster) loci additively offers breeders a tool to tailor phenology more precisely to specific environments. However, the most critical period for yield determination in pulses is podding, not flowering (Sadras and Dreccer 2015; Cerrudo and Naeve 2025), and the time from flowering to podding can vary substantially, weakening the link between flowering and yield (Gimenez et al. 2024).

## Conclusions

Future work should build on these results by expanding NIL evaluations into a broader range of environments, including later sowings where terminal stress is more intense, higher-yielding conditions, and incorporating detailed physiological measurements of radiation-use efficiency, harvest index, and reproductive abortion. For breeding and agronomic relevance, phenological research in chickpea should focus on the flowering-to-podding interval. 

## Supplementary information

Below is the link to the electronic supplementary material.

Supplementary Material 1

Supplementary Material 2

## Data Availability

The data that support the findings of this study are available from the authors upon request.
