# Supplementary material for: Genetic interactions among flowering loci influence phenology, vigour, and yield in chickpea
Source: Mol Breed. 2026 Aug 5;46(8):78. doi: 10.1007/s11032-026-01701-5 (PMC13442789; doi:10.1007/s11032-026-01701-5)
Supplement: Supplementary file 1 [file 11032_2026_1701_MOESM1_ESM.pdf]

Genetic interactions among flowering loci influence phenology, vigour, and yield in chickpea

Molecular Breeding

R. Ortega Martinez[ab], L. Lake[cde], J.E. Hayes[d], J. L. Weller[ab], R. French[f] , V. O. Sadras[cde]

a *School of Natural Sciences, University of Tasmania, Sandy Bay Campus, Hobart, Tasmania, Australia*

b *ARC Centre of Excellence for Plant Success in Nature and Agriculture, Australia*

c *South Australian Research and Development Institute, Australia*

d *Waite Research Institute, School of Agriculture, Food and Wine, The University of Adelaide, Australia*

e *College of Science and Engineering, Flinders University, Australia*

f *Department of Primary Industries and Regional Development, Merredin, Western Australia, Australia*

Corresponding author: Raul Ortega Martinez. E-mail address: rortega@utas.edu.au.

Supplemental Table 1.

Primer sequences (5' – 3') for Kompetetive Allele Specific Polymorphism (KASP™) markers to characterise alleles at phenology loci *CaELF3a*, *CaLG3a* and *CaLG3b*. Primer sequences for *CaELF3a* were sourced directly from Ridge et al. (2017) and target the deletion verified as conferring loss of function. Sequences for *CaLG3a* were sourced from Nguyen et al. (2022). *CaLG3b* was characterised using four markers spanning a broad genetic interval.

**Marker name**	**Locus**	**Allele-specific primer 1**	**Allele-specific primer 2**	**Common primer**
CaELF3a	*CaELF3a*	GAAGGTGACCAAGTTCATGCTGATGAAGAGAGGGAAAGATGATGAG	GAAGGTCGGAGTCAACGGATTGGATGAAGAGAGGGAAAGATGATGAT	CCTCCTTTCTCCGTATCACCAACAT
Ca3_34805805	*CaLG3a*	GAAGGTGACCAAGTTCATGCTTATTTTTACTTATTTGTTTTGGTATCATCTAC	GAAGGTCGGAGTCAACGGATTATTTTTACTTATTTGTTTTGGTATCATCTAG	TCTAAAAGTAGTTTTAAGTACAATCTTCTA
Ca3_LG3b1	*CaLG3b*	GAAGGTGACCAAGTTCATGCTTTTAGAAGGACTTAAGCATTACCTCAT	GAAGGTCGGAGTCAACGGATTTAGAAGGACTTAAGCATTACCTCAC	CCCTCTATATCTTTTCATGAGATCCTTCTA
Ca3_LG3b2	*CaLG3b*	GAAGGTGACCAAGTTCATGCTCAAATAAGTAAACAACAAGCATCTGCC	GAAGGTCGGAGTCAACGGATTGCAAATAAGTAAACAACAAGCATCTGCT	CACGAGCAAAACTCATTGTTTAAACCGTT
Ca3_LG3b3	*CaLG3b*	GAAGGTGACCAAGTTCATGCTATAATAGTTCCCATTAATACTGAAACTGTAATA	GAAGGTCGGAGTCAACGGATTATAGTTCCCATTAATACTGAAACTGTAATG	CTTGATTACAGATAAAGATAAAGGGCACAA
Ca3_LG3b4	*CaLG3b*	GAAGGTGACCAAGTTCATGCTGAAGAAAAGCAGTTTTTCAAAGCTGGT	GAAGGTCGGAGTCAACGGATTGAAGAAAAGCAGTTTTTCAAAGCTGGA	GAAGTTTGACTGAAGGCAATAGACCATAT

Supplemental Table 3.

Mean, Standard deviation (SD), Minimum (Min), Maximum (Max), Range (Ran) and Median (Med) values for 10 phenological traits in parental lines and Near-Isogenic Lines grown under long-day (LD) and short-day (SD) conditions.Traits include days from emergence to floral initiation (DFI), floral development (DFD), and pod development (DPD); corresponding node-based traits (NFI, NFD, NPD); and developmental intervals between stages (DFDELAY, DPDELAY, NFGAP, NPGAP).

		DFI						DFD						DPD						NFI						NFD						NPD						DFDELAY						DPDELAY						NFGAP						NPGAP					
Material	Cond	Mean	SD	Min	Max	Ran	Med	Mean	SD	Min	Max	Ran	Med	Mean	SD	Min	Max	Ran	Med	Mean	SD	Min	Max	Ran	Med	Mean	SD	Min	Max	Ran	Med	Mean	SD	Min	Max	Ran	Med	Mean	SD	Min	Max	Ran	Med	Mean	SD	Min	Max	Ran	Med	Mean	SD	Min	Max	Ran	Med	Mean	SD	Min	Max	Ran	Med
CaLG3a1_G1	LD	34.62	0.92	33	36	3	35	34.62	0.92	33	36	3	35	40.38	0.52	40	41	1	40	18.62	0.74	18	20	2	18.5	18.62	0.74	18	20	2	18.5	18.62	0.74	18	20	2	18.5	0	0	0	0	0	0	5.75	0.89	4	7	3	6	0	0	0	0	0	0	0	0	0	0	0	0
CaLG3a1_G1	SD	71.75	8.24	59	82	23	72.5	79.38	4.41	69	83	14	80	86.62	3.78	79	91	12	86	36.5	3.78	31	41	10	36.5	41.38	2.88	35	44	9	41.5	41.62	2.26	37	44	7	41.5	7.62	8.12	0	21	21	5	7.25	1.58	6	10	4	6.5	4.88	5.25	0	13	13	3	0.25	0.71	0	2	2	0
CaLG3a1_G2	LD	34.88	2.17	32	39	7	34	34.88	2.17	32	39	7	34	40.62	2.33	37	44	7	40	18.12	0.83	17	19	2	18	18.12	0.83	17	19	2	18	18.12	0.83	17	19	2	18	0	0	0	0	0	0	5.75	0.71	5	7	2	6	0	0	0	0	0	0	0	0	0	0	0	0
CaLG3a1_G2	SD	73.62	4.14	68	80	12	74	76.88	4.29	68	81	13	78	83.88	3.09	77	86	9	85	35.5	3.55	32	42	10	34.5	38.88	3.18	35	43	8	38.5	39.25	2.92	35	43	8	39	3.25	2.05	0	5	5	4	7	1.77	5	10	5	6	3.38	2.72	0	7	7	4	0.38	0.52	0	1	1	0
CaLG3a1_R1	LD	32.38	2.07	30	36	6	32.5	32.38	2.07	30	36	6	32.5	38.12	2.64	35	42	7	38	16	1.69	14	18	4	16	16	1.69	14	18	4	16	16	1.69	14	18	4	16	0	0	0	0	0	0	5.75	1.04	4	7	3	6	0	0	0	0	0	0	0	0	0	0	0	0
CaLG3a1_R1	SD	69.33	14.56	51	83	32	70	81	12.38	66	98	32	81	86	9.17	76	97	21	89	33.33	8.62	23	42	19	33.5	39.33	8.41	30	50	20	38	38.2	6.83	32	47	15	35	11.67	6.35	2	20	18	12.5	8.4	2.61	6	12	6	8	6	3.29	1	11	10	6	1	1	0	2	2	1
CaLG3a1_R2	LD	32.25	2.05	29	35	6	32	32.25	2.05	29	35	6	32	38.88	2.3	36	42	6	38.5	16.38	1.06	14	17	3	17	16.38	1.06	14	17	3	17	16.38	1.06	14	17	3	17	0	0	0	0	0	0	6.62	0.74	6	8	2	6.5	0	0	0	0	0	0	0	0	0	0	0	0
CaLG3a1_R2	SD	67.75	6.84	57	76	19	69.5	73	8.55	59	80	21	77.5	80.75	6.69	69	88	19	83	35.75	4.92	30	43	13	35	38.62	4.24	32	43	11	38.5	38.88	4.36	32	44	12	38.5	5.25	5.18	0	14	14	5	7.75	3.2	4	13	9	7	2.88	4.05	0	12	12	2	0.25	0.46	0	1	1	0
CaLG3a2_G	LD	35.88	2.17	33	40	7	35.5	35.88	2.17	33	40	7	35.5	41	1.51	39	43	4	40.5	17.75	0.71	16	18	2	18	17.75	0.71	16	18	2	18	17.75	0.71	16	18	2	18	0	0	0	0	0	0	5.12	0.99	3	6	3	5	0	0	0	0	0	0	0	0	0	0	0	0
CaLG3a2_G	SD	48.12	4.88	43	59	16	47.5	51.5	6.89	43	59	16	52	60.38	5.97	54	68	14	60.5	23.25	1.04	22	25	3	23	24.38	2.26	22	27	5	23.5	24.62	2.13	22	27	5	24	3.38	4.69	0	10	10	0	8.88	2.85	6	14	8	9	1.12	1.64	0	4	4	0	0.25	0.46	0	1	1	0
CaLG3a2_R	LD	32.75	2.25	30	37	7	32.5	32.75	2.25	30	37	7	32.5	38.5	2.14	36	42	6	38	17	0.76	16	18	2	17	17	0.76	16	18	2	17	17	0.76	16	18	2	17	0	0	0	0	0	0	5.75	0.89	5	7	2	5.5	0	0	0	0	0	0	0	0	0	0	0	0
CaLG3a2_R	SD	48	3.02	44	52	8	47.5	54	5.45	44	58	14	57	61.25	3.33	55	64	9	62.5	22.5	0.76	22	24	2	22	24.88	2.03	22	28	6	25	25.12	1.73	22	28	6	25	6	4.21	0	11	11	6.5	7.25	2.43	5	11	6	6.5	2.38	2.07	0	6	6	2.5	0.25	0.71	0	2	2	0
CaLG3a3_G1	LD	38.38	1.51	36	40	4	39	38.38	1.51	36	40	4	39	44	1.41	42	45	3	45	20.88	0.64	20	22	2	21	20.88	0.64	20	22	2	21	20.88	0.64	20	22	2	21	0	0	0	0	0	0	5.62	0.52	5	6	1	6	0	0	0	0	0	0	0	0	0	0	0	0
CaLG3a3_G1	SD	63.38	8.18	57	77	20	60	66.38	7.76	59	80	21	64.5	72.75	7.59	66	86	20	69.5	32.62	3.16	30	39	9	31	33.25	3.28	31	39	8	32	34.12	3.04	31	39	8	33.5	3	3.46	0	7	7	1.5	6.38	1.69	3	8	5	7	0.62	0.92	0	2	2	0	0.88	1.36	0	3	3	0
CaLG3a3_G2	LD	38	1.51	36	40	4	38.5	38	1.51	36	40	4	38.5	44.12	1.36	42	45	3	45	20.25	0.89	19	22	3	20	20.25	0.89	19	22	3	20	20.25	0.89	19	22	3	20	0	0	0	0	0	0	6.12	0.99	5	8	3	6	0	0	0	0	0	0	0	0	0	0	0	0
CaLG3a3_G2	SD	66.5	10.8	54	82	28	65.5	69.75	8.22	60	82	22	66	77.75	5.85	72	88	16	75	32.25	4.2	24	37	13	33.5	34.38	1.69	32	37	5	34.5	34.38	1.69	32	37	5	34.5	3.25	4.43	0	12	12	1	8	3.25	3	12	9	9.5	2.12	3.44	0	10	10	0.5	0	0	0	0	0	0
CaLG3a3_R1	LD	37.43	5.71	32	47	15	35	37.43	5.71	32	47	15	35	45.14	7.63	38	56	18	40	19.57	1.51	17	21	4	19	19.57	1.51	17	21	4	19	19.86	1.95	17	23	6	19	0	0	0	0	0	0	7.71	2.56	5	11	6	6	0	0	0	0	0	0	0.29	0.76	0	2	2	0
CaLG3a3_R1	SD	70.38	5.45	62	77	15	70.5	71.88	6.03	62	80	18	72.5	79.88	5.08	71	86	15	80.5	36.12	1.96	33	39	6	36	37.38	2.33	33	40	7	37	37.88	2.23	34	40	6	38	1.5	1.41	0	3	3	1.5	8	1.93	5	11	6	8.5	1.25	1.28	0	3	3	1	0.5	1.07	0	3	3	0
CaLG3a3_R2	LD	34.88	2.42	32	39	7	34.5	34.88	2.42	32	39	7	34.5	42.25	4.03	38	50	12	42.5	18.25	0.89	17	19	2	18.5	18.25	0.89	17	19	2	18.5	18.38	1.06	17	20	3	18.5	0	0	0	0	0	0	7.38	1.92	5	11	6	7	0	0	0	0	0	0	0.12	0.35	0	1	1	0
CaLG3a3_R2	SD	71.33	6.41	63	80	17	72	73.33	6.41	63	80	17	74.5	80.33	4.93	72	85	13	82	36.5	1.64	34	38	4	37	37.17	2.48	34	40	6	37	37.33	2.5	34	40	6	37.5	2	3.35	0	8	8	0	7	1.79	4	9	5	7.5	0.67	1.03	0	2	2	0	0.17	0.41	0	1	1	0
CaLG3a4_G1	LD	34.25	2.05	31	38	7	34.5	34.25	2.05	31	38	7	34.5	39.38	1.92	36	42	6	40	15.62	0.52	15	16	1	16	15.62	0.52	15	16	1	16	15.62	0.52	15	16	1	16	0	0	0	0	0	0	5.12	0.64	4	6	2	5	0	0	0	0	0	0	0	0	0	0	0	0
CaLG3a4_G1	SD	39.5	2.27	38	43	5	38	39.5	2.27	38	43	5	38	48.88	6.06	44	59	15	46	19.38	0.92	18	21	3	19	19.38	0.92	18	21	3	19	19.75	1.58	18	23	5	19	0	0	0	0	0	0	9.38	3.85	6	16	10	8	0	0	0	0	0	0	0.38	0.74	0	2	2	0
CaLG3a4_G2	LD	33.88	1.13	32	35	3	34	33.88	1.13	32	35	3	34	39.38	0.92	38	41	3	39	16.62	0.52	16	17	1	17	16.62	0.52	16	17	1	17	16.62	0.52	16	17	1	17	0	0	0	0	0	0	5.5	0.76	4	6	2	6	0	0	0	0	0	0	0	0	0	0	0	0
CaLG3a4_G2	SD	39.12	1.55	37	41	4	40	39.12	1.55	37	41	4	40	46.12	2.53	42	49	7	46	20.38	0.74	20	22	2	20	20.38	0.74	20	22	2	20	20.38	0.74	20	22	2	20	0	0	0	0	0	0	7	1.41	5	9	4	7	0	0	0	0	0	0	0	0	0	0	0	0
CaLG3a4_R1	LD	32.88	2.42	31	37	6	31.5	32.88	2.42	31	37	6	31.5	37.62	2.88	35	42	7	36	14.5	0.53	14	15	1	14.5	14.5	0.53	14	15	1	14.5	14.5	0.53	14	15	1	14.5	0	0	0	0	0	0	4.75	0.71	4	6	2	5	0	0	0	0	0	0	0	0	0	0	0	0
CaLG3a4_R1	SD	35.38	4.41	31	45	14	34	35.38	4.41	31	45	14	34	42.75	5.47	39	55	16	41	16.75	1.75	16	21	5	16	16.75	1.75	16	21	5	16	16.75	1.75	16	21	5	16	0	0	0	0	0	0	7.38	1.41	6	10	4	7.5	0	0	0	0	0	0	0	0	0	0	0	0
CaLG3a4_R2	LD	31.57	1.81	29	34	5	31	31.57	1.81	29	34	5	31	36.43	2.07	33	39	6	36	15	0.58	14	16	2	15	15	0.58	14	16	2	15	15	0.58	14	16	2	15	0	0	0	0	0	0	4.86	0.38	4	5	1	5	0	0	0	0	0	0	0	0	0	0	0	0
CaLG3a4_R2	SD	35.12	2.7	30	38	8	36	35.12	2.7	30	38	8	36	42.25	3.69	36	48	12	42.5	16.62	1.19	15	19	4	16.5	16.62	1.19	15	19	4	16.5	16.62	1.19	15	19	4	16.5	0	0	0	0	0	0	7.12	1.89	5	10	5	6.5	0	0	0	0	0	0	0	0	0	0	0	0
CaLG3b1_G	LD	34.5	3.7	30	39	9	34.5	34.5	3.7	30	39	9	34.5	40.25	4.11	35	45	10	40.5	14.75	0.5	14	15	1	15	14.75	0.5	14	15	1	15	14.75	0.5	14	15	1	15	0	0	0	0	0	0	5.75	0.5	5	6	1	6	0	0	0	0	0	0	0	0	0	0	0	0
CaLG3b1_G	SD	39	5.35	34	45	11	38.5	43.75	13.96	34	64	30	38.5	54.5	15.93	41	72	31	52.5	15.75	0.5	15	16	1	16	18.5	5	16	26	10	16	19.75	4.79	16	26	10	18.5	4.75	9.5	0	19	19	0	10.75	7.54	6	22	16	7.5	2.75	5.5	0	11	11	0	1.25	2.5	0	5	5	0
CaLG3b1_R	LD	32.5	1.73	30	34	4	33	32.5	1.73	30	34	4	33	38.25	1.71	36	40	4	38.5	15.5	1	14	16	2	16	15.5	1	14	16	2	16	15.5	1	14	16	2	16	0	0	0	0	0	0	5.75	1.26	4	7	3	6	0	0	0	0	0	0	0	0	0	0	0	0
CaLG3b1_R	SD	52	4.32	48	58	10	51	60.5	4.36	58	67	9	58.5	67	2.94	64	71	7	66.5	22.75	2.36	21	26	5	22	26.5	1.73	25	29	4	26	26.5	1.73	25	29	4	26	8.5	2.08	6	11	5	8.5	6.5	1.91	4	8	4	7	3.75	0.96	3	5	2	3.5	0	0	0	0	0	0
CaLG3b2_G	LD	36.25	0.5	36	37	1	36	36.25	0.5	36	37	1	36	43	0.82	42	44	2	43	18.5	1.29	17	20	3	18.5	18.5	1.29	17	20	3	18.5	18.5	1.29	17	20	3	18.5	0	0	0	0	0	0	6.75	0.96	6	8	2	6.5	0	0	0	0	0	0	0	0	0	0	0	0
CaLG3b2_G	SD	68.25	8.1	59	76	17	69	69.25	6.7	63	76	13	69	79.5	4.2	75	84	9	79.5	32	2.58	29	35	6	32	32.75	1.71	31	35	4	32.5	32.75	1.71	31	35	4	32.5	1	2	0	4	4	0	10.25	2.63	8	13	5	10	0.75	1.5	0	3	3	0	0	0	0	0	0	0
CaLG3b2_R	LD	39.25	1.5	37	40	3	40	39.25	1.5	37	40	3	40	46.25	1.5	44	47	3	47	20.75	0.5	20	21	1	21	20.75	0.5	20	21	1	21	20.75	0.5	20	21	1	21	0	0	0	0	0	0	7	0	7	7	0	7	0	0	0	0	0	0	0	0	0	0	0	0
CaLG3b2_R	SD	70.75	4.79	67	77	10	69.5	71.25	5.68	67	79	12	69.5	82.25	4.72	78	89	11	81	33.25	2.22	31	36	5	33	33.75	3.1	31	38	7	33	36.5	4.04	31	40	9	37.5	0.5	1	0	2	2	0	11	3.83	6	14	8	12	0.5	1	0	2	2	0	2.75	2.22	0	5	5	3
CaLG3b5_G1	LD	35.62	2.62	33	41	8	35	35.62	2.62	33	41	8	35	40.75	2.19	38	45	7	40	15.75	0.71	15	17	2	16	15.75	0.71	15	17	2	16	15.75	0.71	15	17	2	16	0	0	0	0	0	0	5.12	0.64	4	6	2	5	0	0	0	0	0	0	0	0	0	0	0	0
CaLG3b5_G1	SD	49.88	4.19	44	57	13	50.5	53.12	6.88	44	60	16	55	61.75	8.1	52	77	25	61.5	22.12	1.55	19	24	5	22.5	23.25	2.43	19	26	7	24	23.75	2.6	19	27	8	24.5	3.25	4.03	0	9	9	1	8.62	3.7	5	17	12	8	1.12	1.36	0	3	3	0.5	0.5	0.53	0	1	1	0.5
CaLG3b5_G2	LD	34.62	2.45	31	38	7	35	34.62	2.45	31	38	7	35	40.5	2.56	36	45	9	40.5	15.38	0.52	15	16	1	15	15.38	0.52	15	16	1	15	15.38	0.52	15	16	1	15	0	0	0	0	0	0	5.88	0.99	5	7	2	5.5	0	0	0	0	0	0	0	0	0	0	0	0
CaLG3b5_G2	SD	42.5	8.33	34	57	23	39.5	42.88	9.11	34	60	26	39.5	56.38	10.01	44	66	22	60.5	18.75	3.06	15	23	8	18	19	3.51	15	25	10	18	21.62	5.04	15	29	14	23	0.38	1.06	0	3	3	0	13.5	6.39	6	23	17	12	0.25	0.71	0	2	2	0	2.62	3.42	0	9	9	1
CaLG3b5_R1	LD	36.12	2.23	33	40	7	36	36.12	2.23	33	40	7	36	43.12	4.67	39	54	15	42	17.62	0.52	17	18	1	18	17.62	0.52	17	18	1	18	17.75	0.46	17	18	1	18	0	0	0	0	0	0	7	2.93	5	14	9	6	0	0	0	0	0	0	0.12	0.35	0	1	1	0
CaLG3b5_R1	SD	66.5	8.42	50	79	29	68	68	9.06	50	81	31	68	76.5	7.82	60	87	27	78	28.5	2.39	24	32	8	28.5	29.25	2.55	24	33	9	30	29.25	2.55	24	33	9	30	1.5	2.33	0	6	6	0	8.5	2.83	6	14	8	8	0.75	1.16	0	3	3	0	0	0	0	0	0	0
CaLG3b5_R2	LD	34.88	2.03	32	37	5	35	34.88	2.03	32	37	5	35	42	3.55	39	50	11	41.5	16.25	0.46	16	17	1	16	16.25	0.46	16	17	1	16	16.25	0.46	16	17	1	16	0	0	0	0	0	0	7.12	4.05	5	17	12	6	0	0	0	0	0	0	0	0	0	0	0	0
CaLG3b5_R2	SD	54.25	12.02	37	72	35	51.5	64.5	11.82	44	79	35	66.5	78.12	12.38	59	91	32	82	23.62	4.53	16	32	16	24	27.38	4.84	21	35	14	26	30.12	5.74	23	38	15	29.5	10.25	13.12	0	31	31	4.5	13.62	5.55	7	24	17	12	3.75	4.8	0	13	13	2	2.75	3.11	0	9	9	2.5
CaLG3b6_G1	LD	34.12	1.36	32	36	4	34.5	34.12	1.36	32	36	4	34.5	40.5	2.2	38	44	6	40.5	16.62	0.52	16	17	1	17	16.62	0.52	16	17	1	17	16.62	0.52	16	17	1	17	0	0	0	0	0	0	6.38	1.06	5	8	3	6	0	0	0	0	0	0	0	0	0	0	0	0
CaLG3b6_G1	SD	77.88	4.19	75	88	13	76.5	81.88	2.64	80	88	8	81	89.12	3.44	86	97	11	88.5	38.12	3.04	35	44	9	37	41	2.14	38	44	6	41	41.12	2.36	38	45	7	41	4	1.77	0	6	6	4	7.25	1.28	6	9	3	7	2.88	2.03	0	6	6	2.5	0.12	0.35	0	1	1	0
CaLG3b6_G2	LD	32.25	1.04	31	34	3	32	32.25	1.04	31	34	3	32	38.5	1.51	36	41	5	38	16.5	0.76	15	17	2	17	16.5	0.76	15	17	2	17	16.5	0.76	15	17	2	17	0	0	0	0	0	0	6.25	0.89	5	7	2	6.5	0	0	0	0	0	0	0	0	0	0	0	0
CaLG3b6_G2	SD	74.88	9.19	56	90	34	76	80	8.12	62	91	29	81	88.12	5.14	79	97	18	88	36	5.95	23	44	21	36.5	39.88	5.3	28	46	18	41	40.62	4.31	31	46	15	41	5.12	1.96	1	8	7	5	8.12	3.91	5	17	12	6.5	3.88	1.36	2	6	4	3.5	0.75	1.16	0	3	3	0
CaLG3b6_R1	LD	35.5	1.41	34	38	4	35	35.5	1.41	34	38	4	35	42	1.51	40	44	4	41.5	18	0.76	17	19	2	18	18	0.76	17	19	2	18	18	0.76	17	19	2	18	0	0	0	0	0	0	6.5	0.76	6	8	2	6	0	0	0	0	0	0	0	0	0	0	0	0
CaLG3b6_R1	SD	78.4	3.05	75	82	7	78	80	2.92	75	82	7	81	87.4	3.65	83	92	9	87	40.6	1.52	39	43	4	40	42	1	41	43	2	42	42	1	41	43	2	42	1.6	2.07	0	5	5	1	7.4	2.7	3	10	7	8	1.4	1.34	0	3	3	2	0	0	0	0	0	0
CaLG3b6_R2	LD	35.75	1.49	34	39	5	35.5	35.75	1.49	34	39	5	35.5	41.12	0.83	40	42	2	41	18.62	1.3	17	21	4	19	18.62	1.3	17	21	4	19	18.62	1.3	17	21	4	19	0	0	0	0	0	0	5.38	1.06	3	6	3	6	0	0	0	0	0	0	0	0	0	0	0	0
CaLG3b6_R2	SD	76.12	5.46	67	82	15	77.5	78.88	4.73	69	84	15	81	85.5	3.78	78	91	13	86.5	38.38	2	35	41	6	38.5	40.25	2.12	37	43	6	40	40.62	1.77	38	43	5	40.5	2.75	2.66	0	8	8	2	6.62	1.3	5	9	4	6	1.88	1.64	0	4	4	1.5	0.38	0.52	0	1	1	0
CaLG3b7_G1	LD	36	2.98	33	41	8	35	36	2.98	33	41	8	35	41.5	2.78	39	46	7	40.5	16.62	0.52	16	17	1	17	16.62	0.52	16	17	1	17	16.62	0.52	16	17	1	17	0	0	0	0	0	0	5.5	0.53	5	6	1	5.5	0	0	0	0	0	0	0	0	0	0	0	0
CaLG3b7_G1	SD	46.62	2.97	43	51	8	46.5	46.62	2.97	43	51	8	46.5	55.38	2.13	52	59	7	55.5	22.38	0.52	22	23	1	22	22.38	0.52	22	23	1	22	22.62	0.52	22	23	1	23	0	0	0	0	0	0	8.75	2.38	5	12	7	8.5	0	0	0	0	0	0	0.25	0.46	0	1	1	0
CaLG3b7_G2	LD	35.12	1.81	33	38	5	35	35.12	1.81	33	38	5	35	40.75	1.67	39	43	4	41	16.88	0.35	16	17	1	17	16.88	0.35	16	17	1	17	16.88	0.35	16	17	1	17	0	0	0	0	0	0	5.62	0.52	5	6	1	6	0	0	0	0	0	0	0	0	0	0	0	0
CaLG3b7_G2	SD	43.38	1.3	42	46	4	43	43.38	1.3	42	46	4	43	51.5	1.93	50	55	5	51	23	0.76	22	24	2	23	23	0.76	22	24	2	23	23	0.76	22	24	2	23	0	0	0	0	0	0	8.12	0.99	7	10	3	8	0	0	0	0	0	0	0	0	0	0	0	0
CaLG3b7_R1	LD	37.62	0.92	36	39	3	38	37.62	0.92	36	39	3	38	42.88	1.36	41	44	3	43.5	18.12	0.35	18	19	1	18	18.12	0.35	18	19	1	18	18.12	0.35	18	19	1	18	0	0	0	0	0	0	5.25	0.71	4	6	2	5	0	0	0	0	0	0	0	0	0	0	0	0
CaLG3b7_R1	SD	45.62	2.33	44	49	5	44	45.62	2.33	44	49	5	44	54.62	2.5	52	58	6	54	23.62	1.19	22	26	4	23.5	23.62	1.19	22	26	4	23.5	23.75	1.16	22	26	4	24	0	0	0	0	0	0	9	2.14	7	14	7	8.5	0	0	0	0	0	0	0.12	0.35	0	1	1	0
CaLG3b7_R2	LD	37.88	1.55	36	40	4	37	37.88	1.55	36	40	4	37	43.38	2.26	41	47	6	43.5	17.25	0.71	16	18	2	17	17.25	0.71	16	18	2	17	17.25	0.71	16	18	2	17	0	0	0	0	0	0	5.5	1.41	4	8	4	5	0	0	0	0	0	0	0	0	0	0	0	0
CaLG3b7_R2	SD	49.5	5.78	43	57	14	50	49.75	6.18	43	59	16	50	58	5.71	51	67	16	58	24.5	1.51	23	27	4	24.5	24.62	1.6	23	27	4	24.5	25.5	1.77	23	28	5	25.5	0.25	0.71	0	2	2	0	8.25	1.39	6	10	4	8	0.12	0.35	0	1	1	0	0.88	0.99	0	3	3	1
Genesis836	LD	33.86	3.02	30	40	10	33	33.86	3.02	30	40	10	33	40.14	3.53	35	47	12	40	15.29	0.49	15	16	1	15	15.29	0.49	15	16	1	15	15.29	0.49	15	16	1	15	0	0	0	0	0	0	6.29	0.76	5	7	2	6	0	0	0	0	0	0	0	0	0	0	0	0
Genesis836	SD	41.75	6.16	35	51	16	41	58.62	7.19	47	66	19	61.5	68.12	8.31	56	76	20	69.5	18.38	2.2	16	22	6	18	24.62	2.62	20	27	7	26	25	2.51	21	27	6	26	16.88	5.36	12	26	14	15.5	9.5	2.78	6	13	7	8.5	6.25	2.31	3	9	6	6	0.38	0.52	0	1	1	0
Rupali	LD	36.86	1.46	34	38	4	37	36.86	1.46	34	38	4	37	42.29	1.6	39	44	5	43	15.86	0.9	15	17	2	16	15.86	0.9	15	17	2	16	15.86	0.9	15	17	2	16	0	0	0	0	0	0	5.43	0.79	5	7	2	5	0	0	0	0	0	0	0	0	0	0	0	0
Rupali	SD	57.14	9.44	48	76	28	55	62	8.41	48	76	28	61	69.86	5.15	66	81	15	69	26	4.08	23	34	11	24	27.29	3.64	23	34	11	27	28.57	3.15	24	34	10	28	4.86	5.3	0	14	14	5	7.86	4.78	4	18	14	7	1.29	1.5	0	4	4	1	1.29	2.56	0	7	7	0

Supplemental Table 4.

Summary of *CalG3a*, *CalG3b*, and *ELF3a* effect on ten phenological traits in chickpea lines grown under long-day (LD) and short-day (SD) photoperiods. For each trait and condition, all possible contrasts were calculated as (*Genesis836* allele value -*Rupali* allele value) across NIL pairs differing for each locus. The table reports the mean difference between contrasts, standard deviation (SD), standard error (SE), and the number of contrasts (Count) peformed for each locus.

	Locus	*CaLG3a*				*CaLG3b*				*ELF3a*			
Trait	Condition	Mean Diff	SD	SE	Count	Mean Diff	SD	SE	Count	Mean Diff	SD	SE	Count
DFI	LD	2.18	0.93	0.26	13	-1.50	1.57	0.42	14	0.45	1.96	0.26	57
	SD	0.71	4.87	1.35	13	-6.15	7.47	2.00	14	25.69	8.70	1.15	57
DFD	LD	2.18	0.93	0.26	13	-1.50	1.57	0.42	14	0.45	1.96	0.26	57
	SD	0.01	4.51	1.25	13	-6.89	9.36	2.50	14	27.09	11.64	1.54	57
DPD	LD	1.64	1.30	0.36	13	-1.80	1.36	0.36	14	1.17	2.29	0.30	57
	SD	0.55	4.77	1.32	13	-6.50	8.85	2.37	14	25.69	11.95	1.58	57
DFDELAY	LD	0.00	0.00	0.00	13	0.00	0.00	0.00	14	0.00	0.00	0.00	57
	SD	-0.71	2.96	0.82	13	-0.75	3.75	1.00	14	1.40	5.09	0.67	57
DPDELAY	LD	-0.54	0.95	0.26	13	-0.30	0.93	0.25	14	0.72	0.81	0.11	57
	SD	0.02	1.29	0.36	13	0.39	2.33	0.62	14	-1.11	2.48	0.33	57
NFI	LD	1.66	0.71	0.20	13	-1.38	0.67	0.18	14	1.90	0.87	0.12	57
	SD	0.29	3.12	0.87	13	-3.28	2.82	0.75	14	14.28	4.02	0.53	57
NFD	LD	1.66	0.71	0.20	13	-1.38	0.67	0.18	14	1.90	0.87	0.12	57
	SD	0.23	2.92	0.81	13	-3.30	3.47	0.93	14	15.71	5.17	0.69	57
NPD	LD	1.60	0.74	0.21	13	-1.39	0.69	0.18	14	1.92	0.91	0.12	57
	SD	0.55	3.00	0.83	13	-3.39	3.03	0.81	14	15.40	4.99	0.66	57
NFGAP	LD	0.00	0.00	0.00	13	0.00	0.00	0.00	14	0.00	0.00	0.00	57
	SD	-0.06	1.20	0.33	13	-0.02	1.62	0.43	14	1.44	2.46	0.33	57
NPGAP	LD	-0.06	0.11	0.03	13	-0.02	0.05	0.01	14	0.02	0.08	0.01	57
	SD	-0.01	0.43	0.12	13	-0.09	1.34	0.36	14	-0.12	1.11	0.15	57
	Contrast:	$LG3a^{Del} - LG3a^{WT}$				$LG3b^{Early} - LG3b^{Late}$				$ELF3a^{WT} - elf3a$			

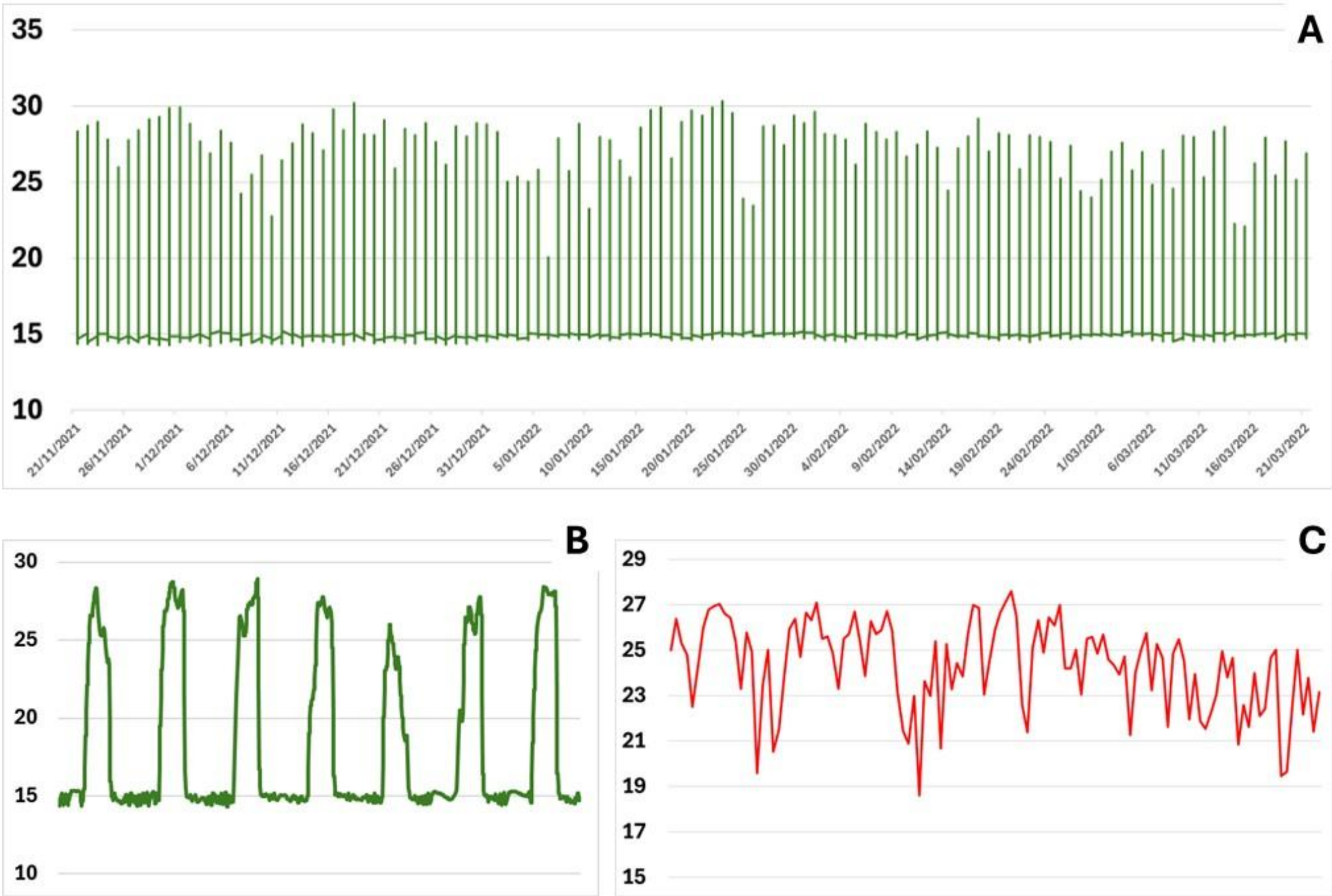

Supplemental Figure 1. Temperature variation (in °C, measured every 10 minutes) experienced by plants grown in controlled environment facilities (Experiment 1) during the scoring period from 21st November 2021 to 21st March 2022 (**A**). Plants were kept inside a phytotron at constant 15 °C for 16 hours/day (4 PM to 8 AM), while subjected to daily variation for 8 hours/day when outside the phytotron (8AM to 4 PM), as shown in (**B**) for a one-week interval, from 21/Nov/2021-12:00 AM to 28/Nov/2021-12:00 AM. **C**) Average daily temperature during the whole scoring period (November 2021-March 2022). Only temperatures recoded between 8AM to 4 PM (plants exposed to variation) were plotted.

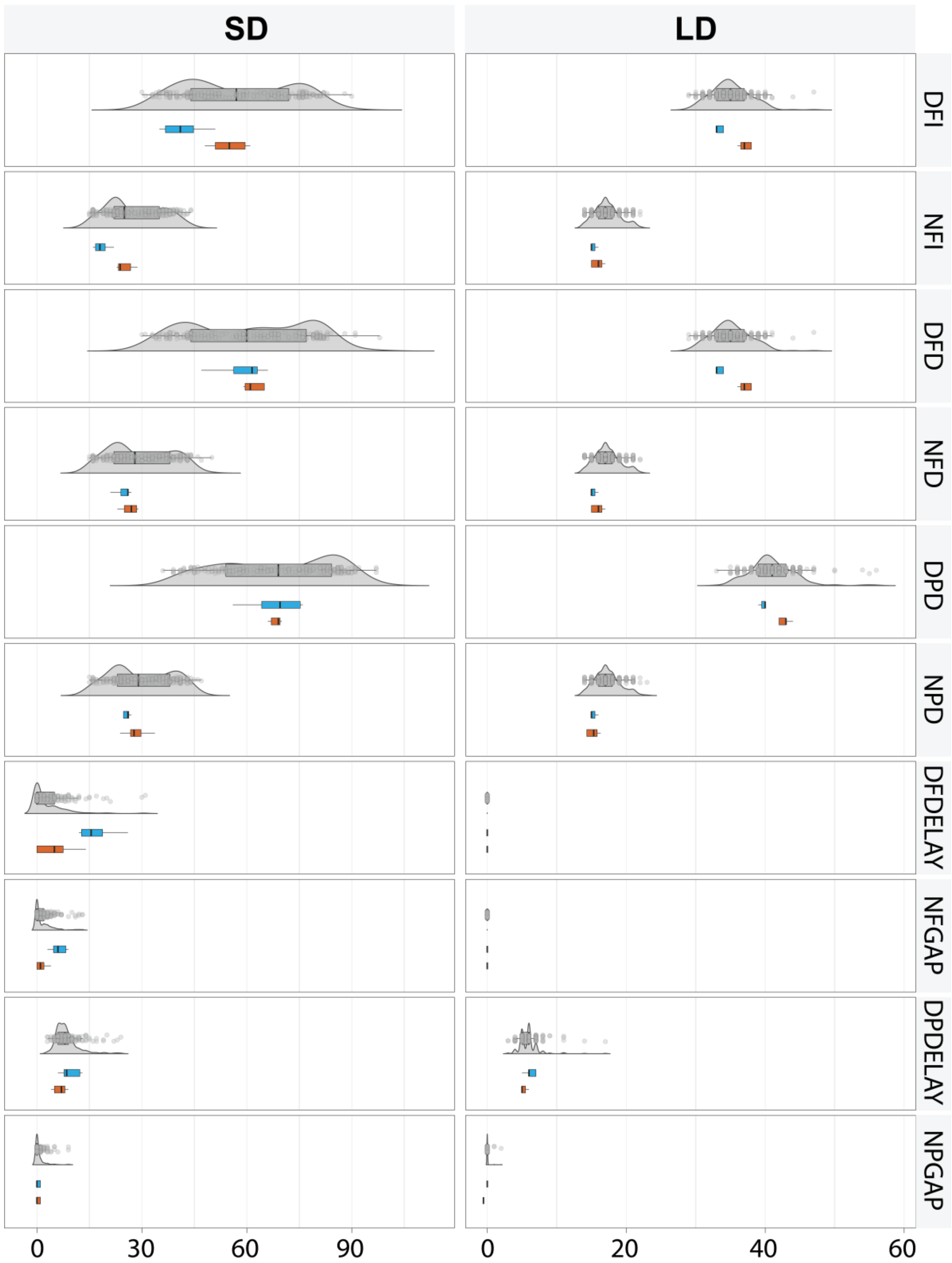

Supplemental Figure 2. Phenotypic variation under long-day (LD, right panel) and short-day (SD, left panel) conditions for chickpea Near-isogenic lines (NILs) and their parental lines. Within each panel, NILs distribution is represented by a grey half violin plot, with overlaid boxplot displaying spread and jittered points representing individual plants. Parents are shown as boxplots only, with Genesis836 in blue and Rupali in orange. The x-axis is the measured value for the trait in each facet, which include node or days to key phenologyical events, as follows: NFI/NFD/NPD = node of flower initiation/flower development/pod development, respectively; DFI/DFD/DPD = their counterparts measured in days. NFGAP/DFDELAY = interval between flower initiation and flower opening (nodes/days); NPGAP/DPDELAY = interval between flower and pod development (nodes/days).

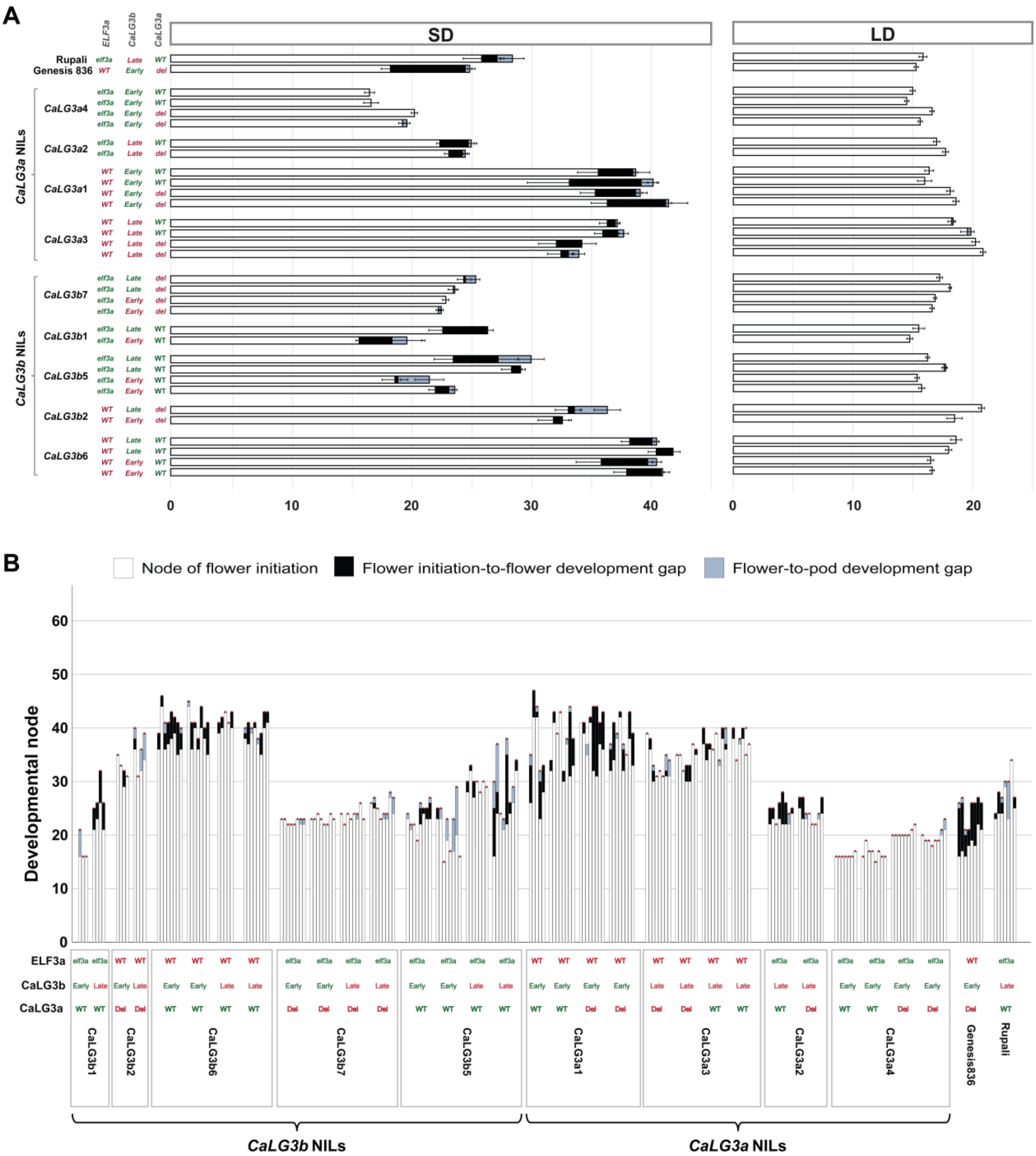

Supplemental Figure 3. (**A**) Variation in the node of flower initiation, flower development, and pod development for the 30 Near-Isogenic Lines and the parental accessions Rupali and Genesis 836 grown under long-day (LD) and short-day (SD) conditions. Bars represent mean values ± standard error. (**B**) Displays the same data but each bar represents an individual plant instead of averages values, to highlight the incidence of aborted flowers (black columns). Allelic composition at *Efl3a*, *CaLG3a*, and *CaLG3b* is shown for all lines, with the colour of the allele indicating whether it promotes (green) or delays (red) phenology in this study.

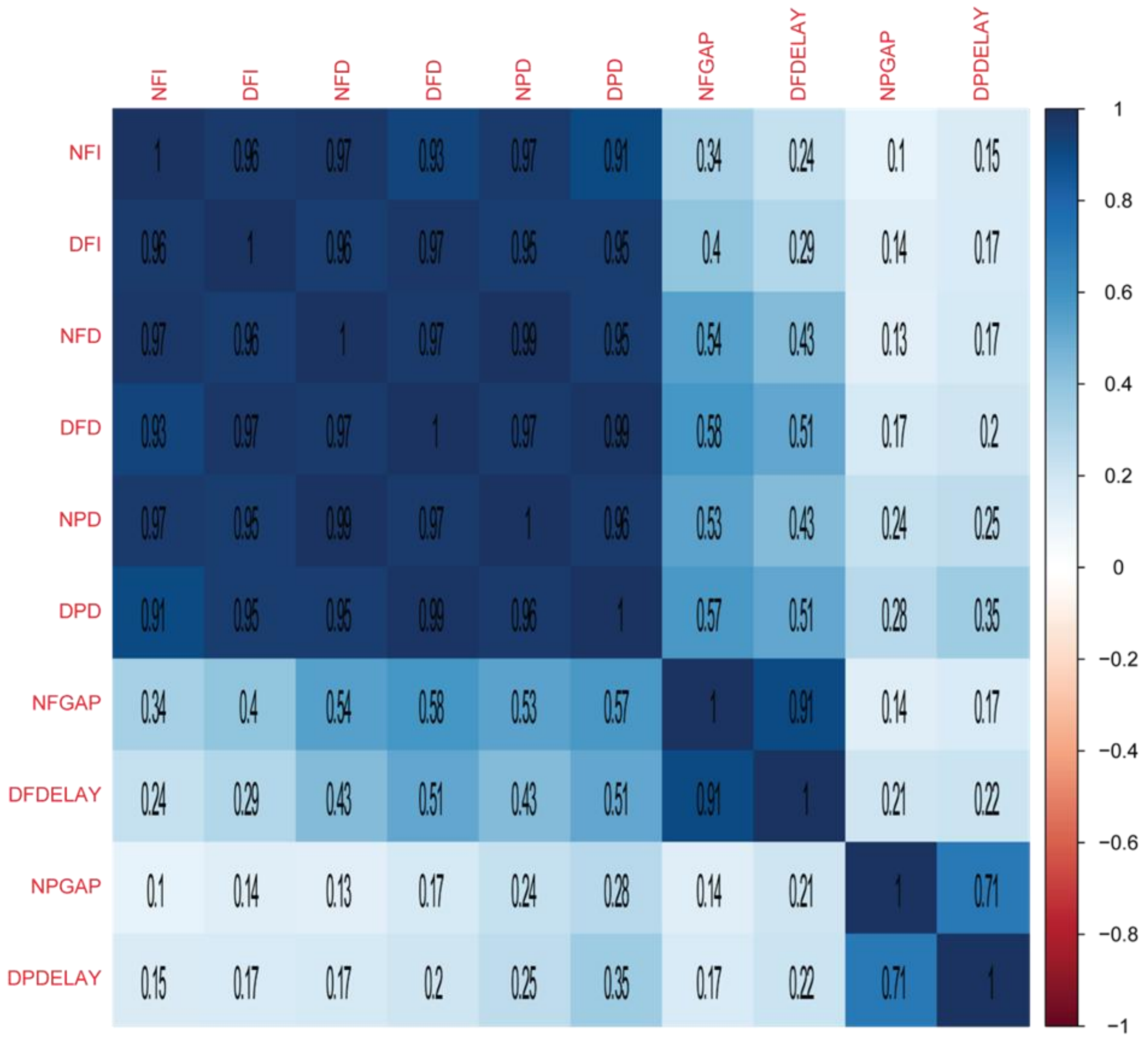

Supplemental Figure 4. Correlation matrix of phenological trait values recorded for NILs and parent genotypes *Genesis836* and *Rupali* in a controlled environment facility under two daylength regimes. Traits include days to flower initiation (DFI), flower development (DFD), pod development (DPD), and node counts (NFI, NFD, NPD), as well as developmental gaps and delays (NFGAP, DFDELAY, NPGAP, DPDELAY). Colour intensity represents Pearson correlation coefficients, ranging from -1 (strong negative correlation, red) to +1 (strong positive correlation, blue).

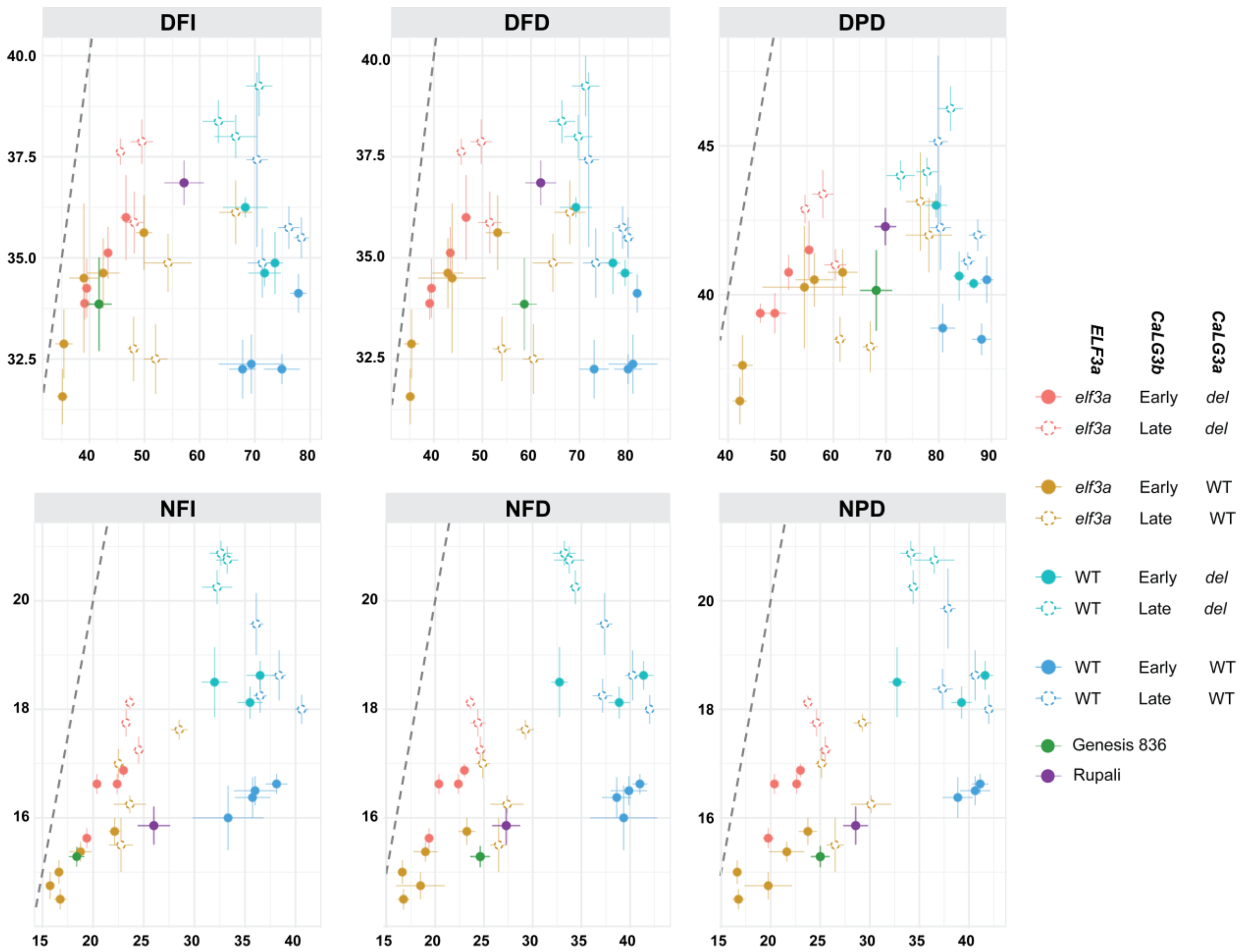

Supplemental Figure 5. Comparison of six phenological traits measured under long-day (LD; Y-axis) and short-day (SD; X-axis) photoperiods in 30 chickpea near-isogenic lines (NILs) and parental controls. Traits include days from emergence to flower initiation (DFI), flower development (DFD) and pod development (DPD), and node positions for flower initiation (NFI), flower development (NFD), and pod development (NPD). Each point represents the mean value for a particular NIL, with vertical and horizontal error bars indicating standard deviation under LD and SD conditions, respectively. Colours and bold/dashed lines denote genotype at *ELF3a*, *CaLG3b*, and *CaLG3a*, as indicated in the legend. The dashed diagonal line represents the 1:1 relationship between LD and SD measurements (photoperiod insensitivity).

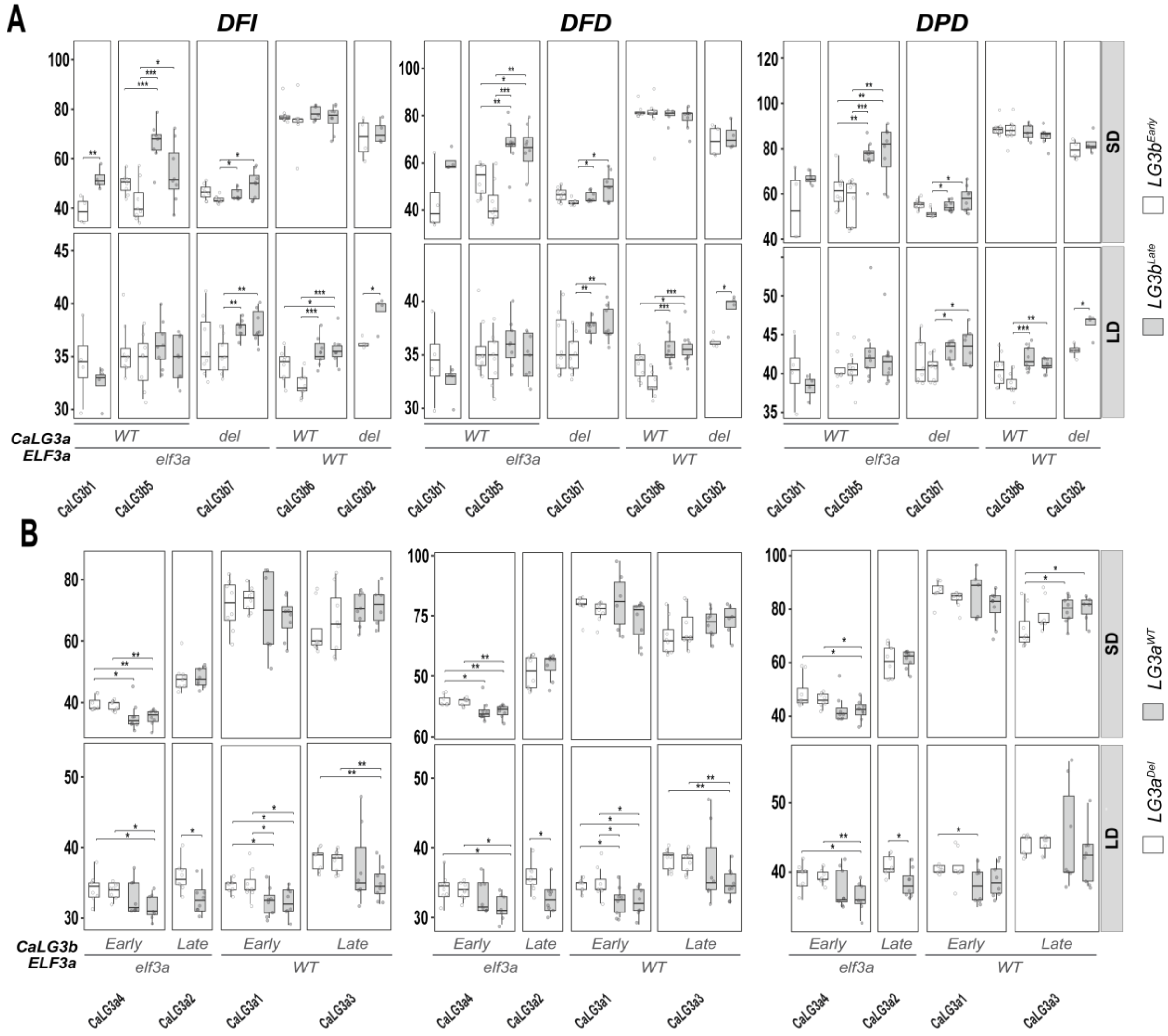

Supplemental Figure 6. Variation in days from emergence to flower initiation (DFI)**,** flower development (DFD) **and** pod development (DPD**)** across Near-Isogenic Lines (NILs) segregating for *CaLG3b* (**A**) or *CaLG3a* (**B**) and grown under long day (LD) or short-day (SD) conditions. The genotype of each NIL for other loci is indicated at the bottom. Grey boxes represent alleles from *Rupali* and white boxes alleles from *Genesis836*. Points represent individual observations and asterisks denote significant differences between allelic classes.

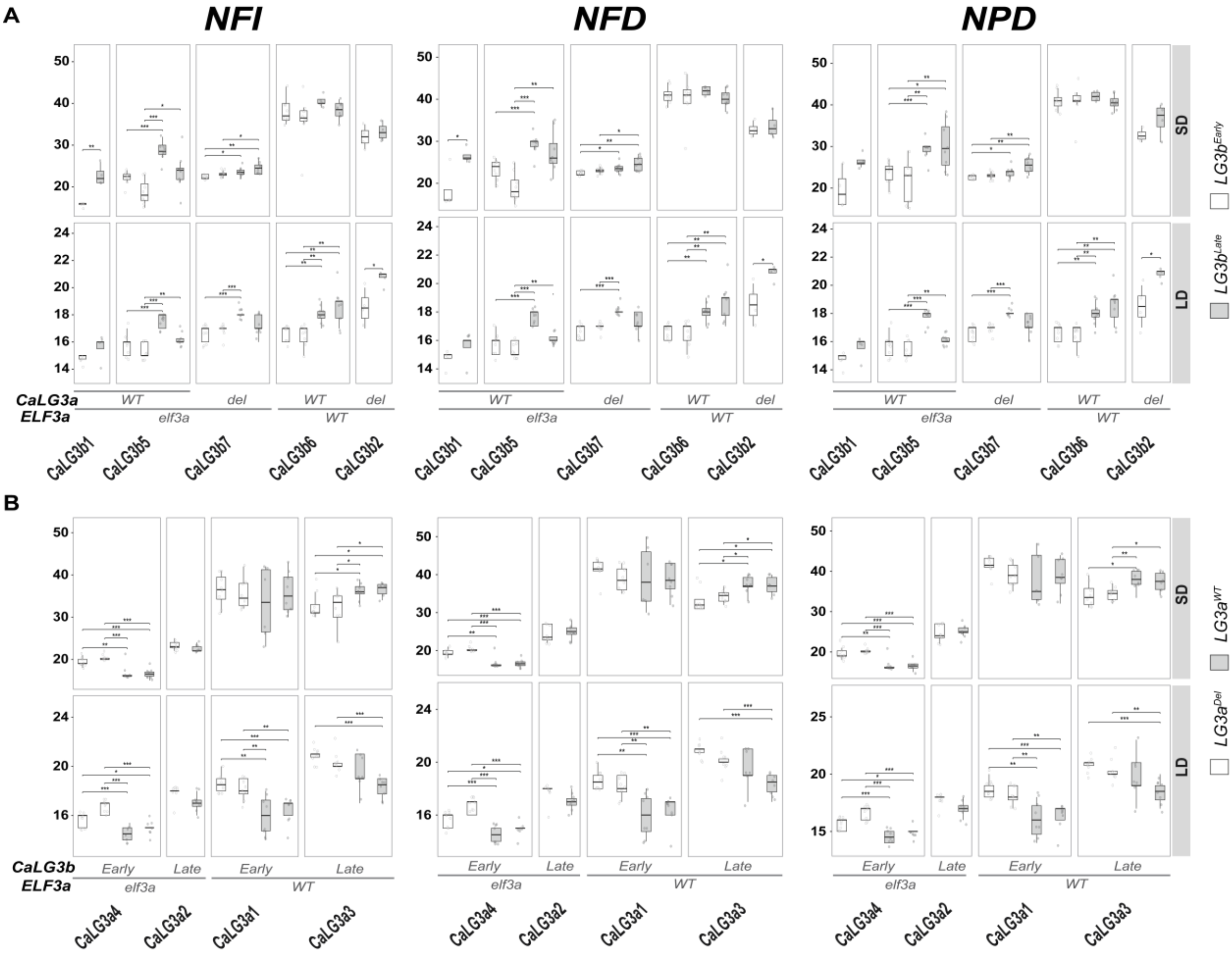

Supplemental Figure 7. Variation in node of flower initiation (NFI), node of flower development (DFD) and node of pod development (DPD) across Near-Isogenic Lines (NILs) segregating for *CaLG3b* (**A**) or *CaLG3a* (**B**) and grown under long day (LD) or short-day (SD) conditions. The genotype of each NIL for other loci is indicated at the bottom. Grey boxes represent alleles from Rupali and white boxes alleles from Genesis 836. Points represent individual observations and asterisks denote significant differences between allelic classes.

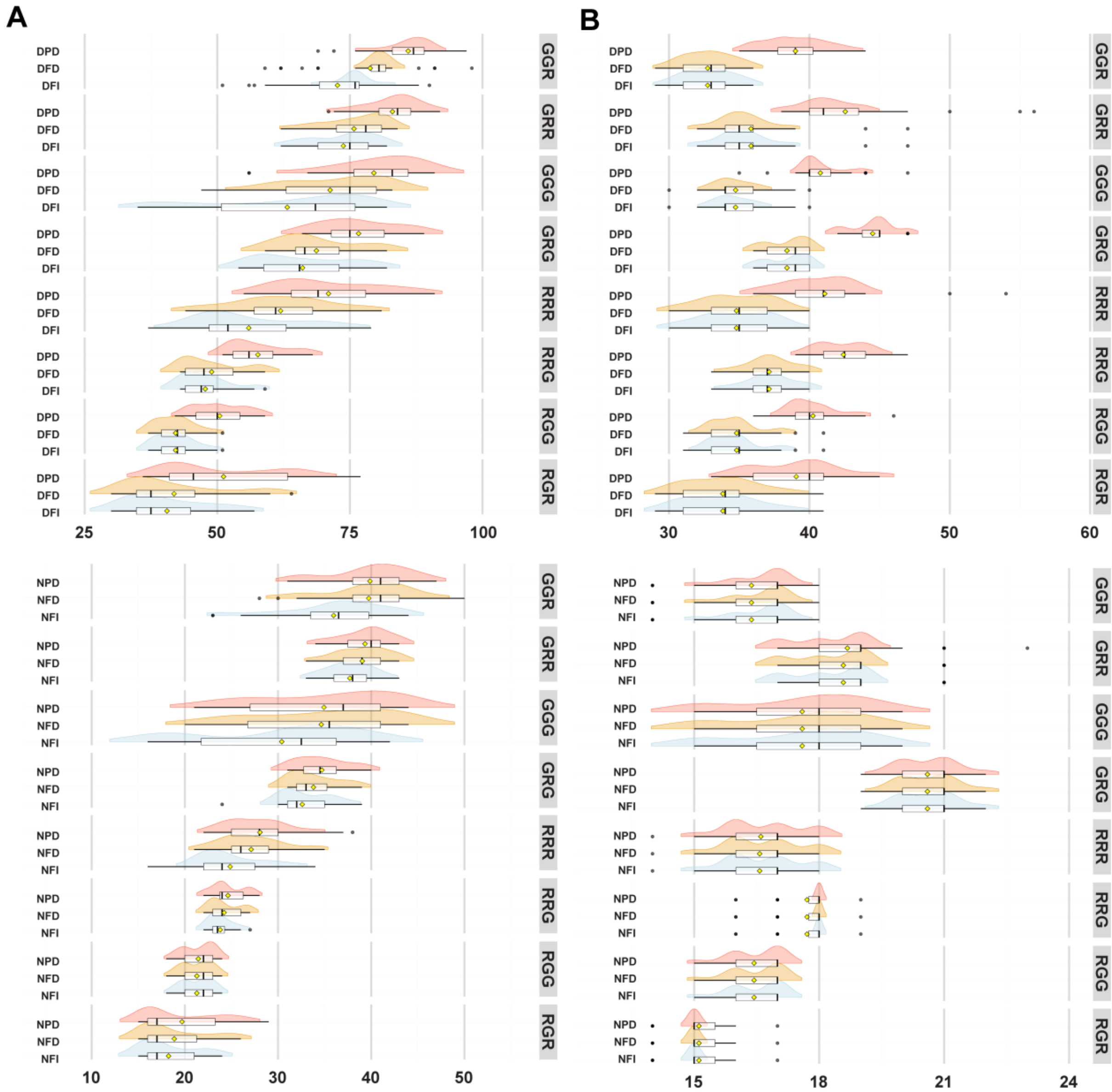

Supplemental Figure 8. Distribution of phenological traits in chickpea Near-Isogenic Lines (NILs) grown under short days (**A**) or long days (**B**). Traits include days flower initiation (blue), flower development (orange) and pod development (red), measured either as days from germination (top panel; DFI, DFD and DPD) or node on the main stem (bottom panel; NFI, NFD and NPD). NILs were grouped by their allelic combination at *ELF3a*, *CaLG3b* and *CaLG3a*, indicated in grey boxes on the right (e.g., GGG, GRG, RRR, where G = Allele from Genesis 836 and R = Allele from Rupali) and sorted by their phenotype under SD. Yellow diamonds in boxplots indicate median values.
